# Laser Ultrasound Super‐Resolution Imaging for Multi‐Parametric Non‐Invasive Volumetric Characterization of Brain Cancer

**DOI:** 10.1002/advs.202502298

**Published:** 2025-09-30

**Authors:** Daniil Nozdriukhin, Yi Chen, Shuxin Lyu, Daniel Razansky, Xosé Luís Deán‐Ben

**Affiliations:** ^1^ Institute for Biomedical Engineering and Institute of Pharmacology and Toxicology Faculty of Medicine University of Zürich Winterthurerstrasse 190 Zurich 8057 Switzerland; ^2^ Institute for Biomedical Engineering Department of Information Technology and Electrical Engineering ETH Zürich Rämistrasse 101 Zurich 8093 Switzerland; ^3^ Institute of Medical Technology Shanxi Medical University Taiyuan 030001 China

**Keywords:** glioblastoma, laser ultrasound imaging, microbubbles, optoacoustic imaging

## Abstract

Accurate characterization of brain tumor heterogeneities and microvascular dynamics remains an unmet need in cancer research driving the development of innovative imaging methods. Here, laser ultrasound super‐resolution imaging (LUSSI) is introduced, a hybrid modality combining laser‐generated ultrasound with optoacoustic (OA) contrast to enable functional volumetric (3D) super‐resolution imaging of vascular structures in vivo. The system integrates a laser ultrasound (LUS) emitter and a spherical array transducer to localize microbubble responses in 3D, further enabling concurrent acquisition of multi‐spectral (multi‐wavelength) OA data. The capabilities of LUSSI to assess tumor heterogeneities are demonstrated in a murine glioblastoma model, including visualization of intra‐tumoral vasculature, identification of compressed vessels, and detection of functional abnormalities such as hemorrhages and hypoxic regions. OA imaging further enables resolving oxygenated and deoxygenated forms of hemoglobin, revealing heterogeneous perfusion within the tumor core. LUSSI is further shown to resolve whole‐brain vascular networks with ≈35 µm resolution and quantify microvascular blood flow and oxygenation, supporting applications in neuroscience. With its non‐invasive, multi‐parametric capabilities and proven compatibility with clinical imaging components, LUSSI offers a promising platform for studying vascular pathology, monitoring therapy, and advancing translational imaging strategies.

## Graphical abstract

Laser ultrasound super‐resolution imaging (LUSSI) leverages laser‐generated ultrasound for functional 3D transcranial imaging of brain tumor vasculature. Combined with optoacoustic tomography, it is demonstrated in a glioblastoma model, revealing structural abnormalities, compressed vessels, hemorrhages, and hypoxic regions. LUSSI provides a non‐invasive platform for investigating vascular pathology and advancing oncology, neuroscience, and translational imaging research.

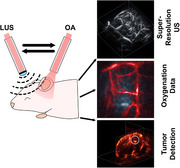

## 1 Introduction

The non‐ionizing nature, proven efficacy, and broad versatility of ultrasound (US) make it an indispensable tool in modern biology and medicine, with applications ranging from preclinical research to clinical diagnostics and therapy.^[^ ^1^, ^2^, ^3^, ^4^, ^5^, ^6^ ^]^ Despite its long history, biomedical US remains a field of innovation, continually advancing through improvements in sensors,^[^ ^4^, ^7^ ^]^ beamforming strategies,^[^ ^8^, ^9^, ^10^ ^]^ new tomographic approaches,^[^ ^11^, ^12^ ^]^ and emerging therapeutic applications such as gene and drug delivery^[^ ^13^, ^14^ ^]^ or neuromodulation.^[^ ^15^, ^16^ ^]^ A significant recent breakthrough has been the development of super‐resolution US imaging, which has elegantly overcome the acoustic diffraction limit of standard pulse‐echo US via individual detection and tracking of microbubbles flowing in blood.^[^ ^17^, ^18^, ^19^ ^]^ Angiographic images are rendered by localizing the positions of flowing microbubbles, resolving otherwise invisible microvasculature with spatial resolution ultimately limited by the signal‐to‐noise ratio (SNR) and the number of detected bubbles.^[^ ^20^ ^]^ Super‐resolution US thus holds strong promise for diagnosing and managing a wide range of diseases linked to microcirculatory changes.^[^ ^21^ ^]^

In parallel, biomedical US is increasingly capitalizing on the advantages of laser light. Laser generation of US, first observed shortly after the invention of the laser,^[^ ^22^ ^]^ provides an alternative to active driving of piezoelectric transducers. In laser ultrasound (LUS), US waves used to interrogate biological tissues are generated at an optical absorber placed within the laser beam path. The efficiency and frequency characteristics of these waves depend on the optical and thermal properties of the absorber. Unlike conventional transducers limited by narrow resonance bandwidths, LUS can generate broadband signals reaching frequencies above 100 MHz for thin films,^[^ ^23^, ^24^, ^25^ ^]^ facilitating both high‐resolution imaging and deep tissue penetration. Fully non‐contact operation, where both US generation and detection are optical, is also possible.^[^ ^26^ ^]^ Another important advantage of LUS is that it enables decoupling of emission and detection pathways, facilitating the implementation of US imaging approaches based on cost‐effective, receive‐only multichannel acquisition systems. Combined with ongoing advances in light‐emitting diodes (LED) and laser diode excitation sources,^[^ ^27^, ^28^, ^29^, ^30^, ^31^ ^]^ the cost of LUS‐based systems could be further significantly reduced. The receive‐only approach not only facilitates high‐speed imaging but also enables hybridization with modalities sensitive to electromagnetic fields, such as magnetic resonance imaging (MRI).^[^ ^32^, ^33^, ^34^ ^]^ LUS then offers low‐cost flexibility for the generation of broadband pulses in disturbance‐prone environments that can enhance the achievable resolution if US sensors with similar detection bandwidth are employed.

Laser generation of US is also the underlying principle of optoacoustic (OA, or photoacoustic) imaging,^[^ ^35^, ^36^, ^37^, ^38^ ^]^ a modality that has become increasingly prominent in biological research. OA imaging offers molecular‐specific optical contrast combined with US‐associated resolution at depths beyond the reach of purely optical techniques. Of particular importance is OA's ability to non‐invasively assess oxygen saturation by exploiting the distinct absorption spectra of oxygenated and deoxygenated hemoglobin,^[^ ^39^, ^40^ ^]^ as demonstrated in multiple ongoing clinical trials.^[^ ^37^, ^41^, ^42^ ^]^ A wide range of OA imaging systems has been developed, spanning microscopic to macroscopic scales.^[^ ^43^, ^44^ ^]^ Because of OA's tomographic nature, maximizing angular coverage is critical to minimize limited‐view artifacts and achieve quantitative imaging at millimeter to centimeter depths.^[^ ^45^ ^]^ Specifically designed arrays of transducers with large angular aperture are increasingly being used by different groups in the field,^[^ ^46^, ^47^, ^48^, ^49^, ^50^ ^]^ and commercial systems based on similar customized US probes have also been essential to spread OA among researchers and clinicians.

The exceptional angiographic imaging capabilities of super‐resolution US and OA imaging make them well‐suited for visualizing the complex vascular networks of the rodent brain and for assessing structural and functional changes in clinically relevant disease models. Both modalities have been widely used as powerful research tools in neuroscience, providing dynamic visualization of brain activity and insights in preclinical models of stroke, neurodegenerative diseases, and brain cancer.^[^ ^51^, ^52^, ^53^, ^54^, ^55^, ^56^, ^57^, ^58^, ^59^, ^60^ ^]^ Combining and co‐registering super‐resolution US and OA imaging further enhances data clarity by integrating the anatomical and structural contrast provided by US with the molecular‐specific optical contrast of OA, particularly in the context of the brain tissue oxygenation.^[^ ^61^, ^62^, ^63^ ^]^ However, despite these advances, brain cancer imaging remains challenging, and reports of in vivo volumetric brain tumor imaging using US and OA techniques are still relatively scarce. This has mainly been done with either *ex vivo* measurements, cranial windows, or 2D‐imaging approaches.^[^ ^64^, ^65^, ^66^, ^67^, ^68^ ^]^ Key technical challenges include 1) the strong frequency‐dependent acoustic attenuation and distortion of the skull, which effectively reduces signal bandwidth; 2) the need for volumetric (3D) imaging to capture tumor heterogeneity without invasive procedures; and 3) the difficulty of co‐registering multi‐parametric data (e.g., vascular structure and oxygenation) in a single platform.

To overcome these challenges, we introduce laser ultrasound super‐resolution imaging (LUSSI), which uniquely integrates three methodological innovations, namely 1) coaxial laser‐generated US excitation and detection covering a suitable bandwidth for precise microbubble tracking despite skull‐induced aberrations; 2) tailored acquisition to achieve concurrent LUS and OA volumetric images without the need for skin or skull removal; and 3) co‐registered super‐resolution vascular mapping and OA‐based oximetry, providing multi‐parametric data for a better characterization of tumor heterogeneities. Motivated by this unique potential for the characterization of brain diseases and the complementary use of short‐pulsed laser sources for LUS and OA, LUSSI can achieve 3D super‐resolution US visualization of the entire murine brain, complemented with an OA contrast to resolve hemoglobin oxygenation. Laser generation of US occurs at the tip of a light guide, coated with carbon, inserted within the central cavity of a spherical array transducer enabling coaxial US excitation and detection of microbubble responses along with concomitant acquisition of OA signals across a sufficiently large angular aperture to achieve accurate tomographic reconstructions. LUSSI is shown to offer a unique non‐invasive and multi‐parametric imaging performance that can effectively overcome the limitations of existing approaches for the assessment of complex heterogeneities of brain tumors in a fully non‐invasive manner. LUSSI, and OA images are shown to complement MRI to provide a comprehensive information potentially enabling the identification of new angiographic and hypoxic markers of clinically relevant tumor models.

## 2 Results

### 2.1 Laser Generation of Ultrasound with Guided Light

**Figure 1:**
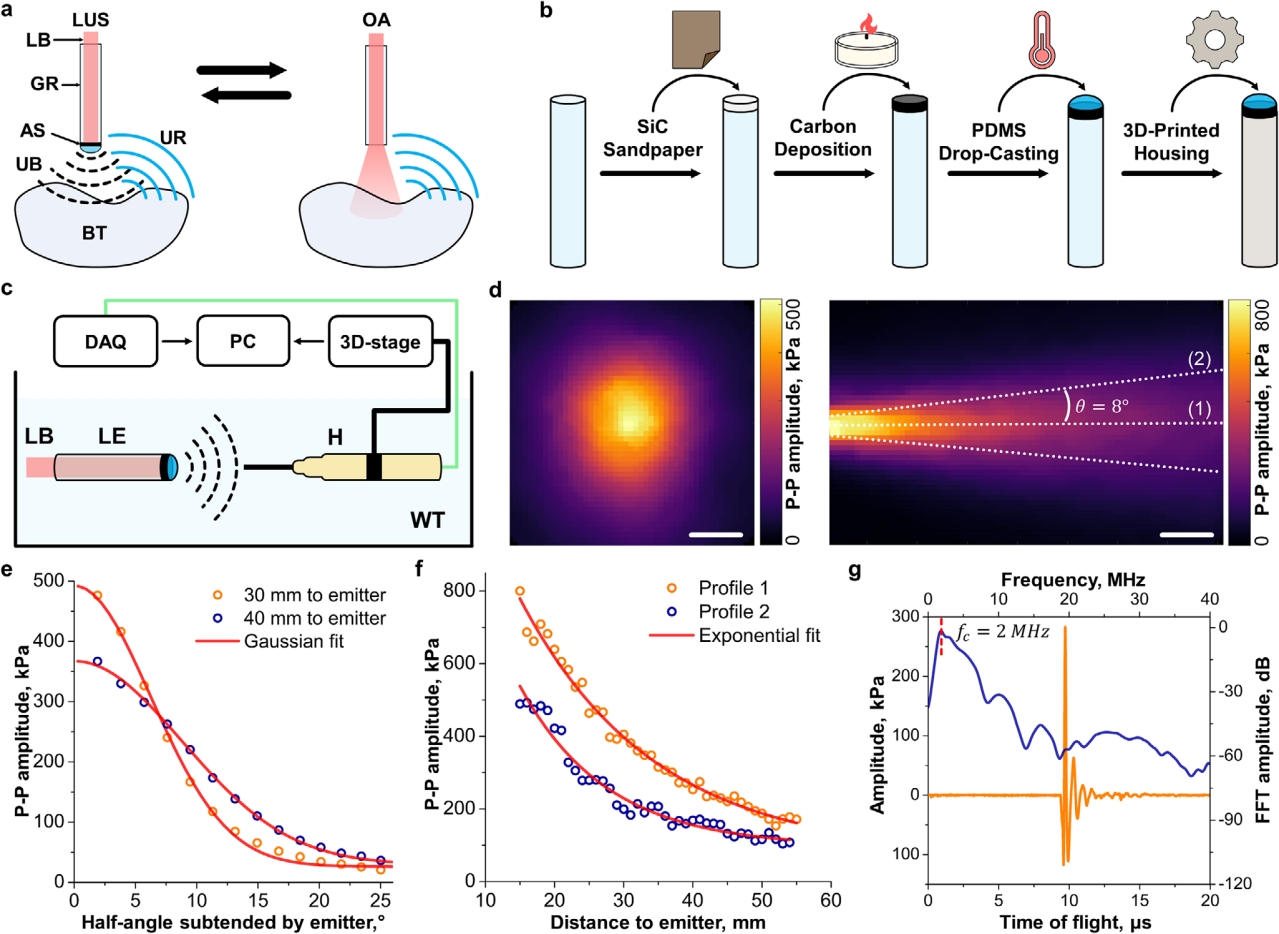
Laser ultrasound (LUS) emitter and generated ultrasound (US) wavefield. a) Switchable excitation of LUS and OA signals in a biological sample with light guided though a glass rod. LB – laser beam, GR – glass rod, AS – absorbing surface, UB – ultrasound beam, UR – ultrasound response, BT – biological tissue. b) Schematic representation of the manufacturing process of the LUS emitter. c) Lay‐out of the experimental set‐up employed for the characterization of the generated US wavefield. DAQ – data acquisition system, PC – personal computer, LB – laser beam, LE – LUS emitter, H – hydrophone, WT – water tank. d) Transverse section of the peak‐to‐peak (P‐P) amplitude of the pressure field at 30 mm distance from the LUS emitter (left) and axial P‐P pressure distribution for the 15–55 mm distance range from it (right). Scalebars – 5 mm. e) P‐P acoustic pressure as a function of the half angle subtended by the LUS emitter at 30 and 40 mm distance. f) P‐P emitted pressure as a function of the distance from the LUS emitter along lines (1) and (2) indicated in panel (d). g) Time‐resolved emitted LUS signal and its frequency content at 30‐mm distance from the LUS emitter.

The use of short‐pulsed lasers for the generation of US waves at light absorbing elements can lead to significant reduction in the complexity of ultrafast US imaging systems.^[^ ^26^ ^]^ Indeed, LUS imaging can be implemented with US transducers and electronics operating solely in receive mode. The development of these components has been fostered by the increasingly prominent role of OA imaging in preclinical studies and recent clinical trials, opening new avenues for the development of hybrid LUS‐OA imaging systems based on sequential excitation of a tissue volume with US and light. Light guiding elements, including optical fibers, fiber bundles, glass rods, or liquid light guides can efficiently deliver light in OA imaging systems and can also be adapted for LUS (**Figure** **1**a). A simple method for depositing an absorbing layer on the output end of a glass rod was devised to produce strong US waves covering a relatively large area of the murine brain (Figure 1b; Figure S1, Supporting Information, see Experimental Section for details). The flat end of the rod was first cleaned to remove organic deposits and subsequently roughened to facilitate attachment of polydimethylsiloxane (PDMS), followed by deposition of carbon soot with a wax candle flame and an additional dome‐shaped PDMS layer. The rod was eventually inserted into a 3D‐printed housing for characterization and imaging experiments. Characterization of the emitted LUS signal was performed by scanning a 1‐mm diameter calibrated needle hydrophone along the axial and transversal directions (Figure 1c, see Experimental Section for details). Peak‐to‐peak US pressure values ≈500 kPa were measured at a distance of 30 mm from the rod surface when exciting it with short laser pulses (≈6 ns, 720 nm) with 14 mJ optical energy. The US intensity can be reduced by adjusting the laser energy to efficiently excite microbubbles without inducing inertial cavitation events that can destroy them.^[^ ^69^ ^]^ The stability of the LUS emitter was assessed by acquiring the generated US signal for 20.000 pulses, showing no evident intensity drop (Figure S1, Supporting Information). Potential PDMS damage was also assessed at the same energy level via optical microscopy (Figure S1, Supporting Information).

The LUS‐emitted beam exhibited a relatively large width at the operating distance of 30–40 mm, with a full width at half maximum (FWHM) of ≈8–14 mm (Figure 1d,e). This corresponds to the focal distance of the spherical array transducer used in subsequent experiments and matches a typical imaging depth of ≈10 mm. Within this range, axial variations in US amplitude were relatively small, showing less than a 25% reduction (Figure 1f). Additionally, as Figure 1g shows, the bandwidth of the emitted LUS signals (7.4 MHz at −30 dB) efficiently covers the detection bandwidth of standard piezoelectric arrays operating at frequencies of several MHz and facilitates excitation of microbubbles with resonances in this frequency range.^[^ ^70^ ^]^

### 2.2 High‐Resolution Co‐Registered Laser Ultrasound and Optoacoustic Imaging

OA imaging exploits the strong, spectrally distinct absorption of hemoglobin to map oxygen saturation within vascular networks, a capability that has driven its growing adoption in both research and clinical applications.^[^ ^37^, ^71^ ^]^ Separately, the introduction of microbubble contrast agents has transformed US into an effective tool for angiography, providing detailed visualization of arteries and veins along with quantitative flow measurements.^[^ ^72^ ^]^ The combination of US and OA imaging leverages their complementary structural and functional strengths,^[^ ^73^ ^]^ with OA adding critical insights into tissue oxygenation at the mesoscopic scale. However, integrating these modalities remains challenging due to differences in operating principles and the types of transducer arrays employed. Specifically, spherical arrays, which maximize angular coverage, are essential for accurate OA imaging, whereas conventional US typically relies on linear or planar arrays. The use of a LUS emitter addresses this limitation by enabling multi‐modal imaging with a spherical array operating solely in reception mode, facilitating direct co‐registration of US and OA data.

**Figure 2:**
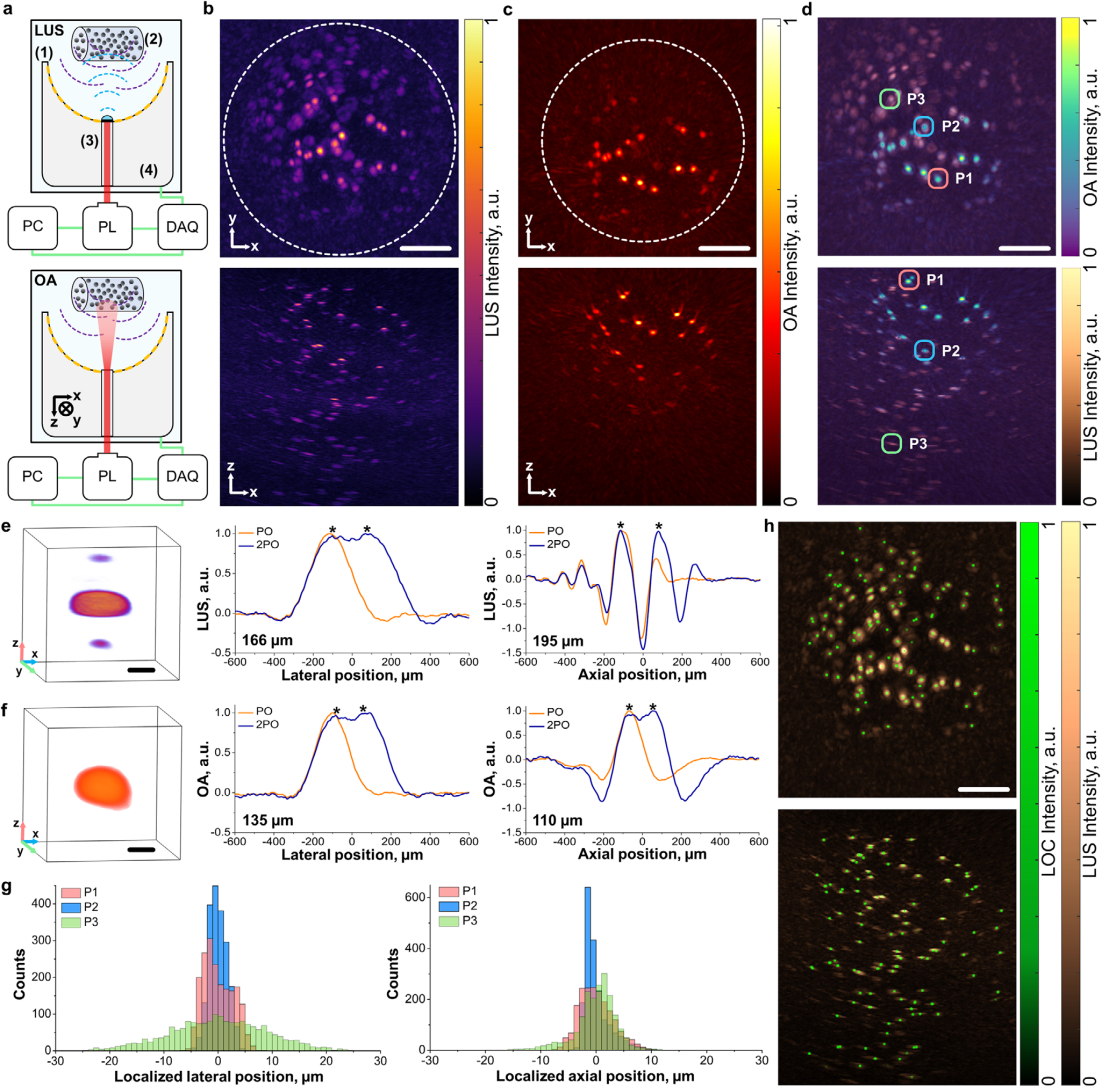
High‐resolution co‐registration of laser ultrasound (LUS) and optoacoustic (OA) images. a) Principal scheme of the experimental set‐up used to acquire LUS (top) and OA (bottom) images of an agar phantom consisting of sparsely distributed 90‐µm black polyethylene spheres. b) Top and lateral maximum intensity projections (MIPs) of the reconstructed LUS image of the phantom. c) Top and lateral MIPs of the reconstructed OA image of the phantom. d) Superimposed LUS and OA MIPs of the phantom. e) 3D view of a microsphere at approximately the central position of the LUS image (left) along with the lateral (middle) and axial (right) profiles of these (orange). Added shifted profiles indicating minimum distinguishable distances are also shown (blue). PO – point object, 2PO – two point objects. f) 3D view of a microsphere at approximately the central position of the OA image (left) along with the lateral (middle) and axial (right) profiles of these (orange). Added shifted profiles indicating minimum distinguishable distances are also shown (blue). g) Histograms of the localized positions in the lateral (left) and axial (right) directions for the points indicated in panel (d) in a sequence of 2000 frames. h) LUS‐based super‐resolution image (LOC, green) built by superimposing the localized points in a sequence of 2000 frames, overlaid on the conventional LUS image. Scalebars – 2 mm (b‐d and h) and 100 µm (e‐f).

The performance of this approach was evaluated by imaging a tissue‐mimicking agar phantom embedded with light‐absorbing 90 µm black polyethylene microspheres (**Figure** **2**a; see Experimental Section for details). LUS imaging was performed using a carbon‐coated emitter placed in the aperture of a sparse spherical array transducer optimized for OA imaging (Figure 2a, top). The microsphere distribution was clearly resolved within an 8 × 8 × 8 mm^3^ volume, comparable to the dimensions of the murine brain (Figure 2b). This imaging volume was primarily limited by the US beam coverage and the transducer detection range. Also, limited‐view artifacts caused noticeable image distortion at larger reconstruction volumes.

OA imaging of the same phantom was conducted by replacing the emitter with a custom‐made fiber bundle (Figure 2a, bottom). The microsphere distribution was similarly resolved, although the OA signal intensity decreased with depth due to optical attenuation within the phantom (Figure 2c). Because both LUS and OA signals were acquired using the same detector array at the same position, the resulting images were naturally co‐registered and could be precisely overlaid (Figure 2d). This co‐registration enables oxygen saturation readings from OA imaging to be accurately assigned to arterioles and venules visualized with LUS, with the large angular aperture critical for achieving quantitative performance.

The achievable axial and lateral resolution for both imaging modes was estimated by analyzing OA and LUS images of a single microsphere positioned near the center of the array (Figure 2e,f; see Experimental Section for details). The acquired signals were filtered from 1 to 8.5 MHz bandwidth, matching the expected frequency content of microbubble echoes and optimizing the field of view for murine brain imaging.^[^ ^74^ ^]^ The high angular coverage of the array enabled an almost isotropic resolution in both modes, with minor variations attributed to acoustic reverberations and frequency‐dependent scattering within the microspheres. These artifacts had minimal impact on the localization accuracy of microsphere centers in LUS images, which provides an estimate of the achievable microbubble localization precision in vivo.

Repeated acquisitions (*n* = 2000) revealed localization uncertainties below 15 µm in all directions for selected points across a depth range equivalent to the dorsal‐ventral extent of the murine brain (Figure 2g). Furthermore, identification of microsphere positions in averaged LUS images, followed by repeated localization using a fixed point spread function (PSF), confirmed that sufficient accuracy was achieved to significantly enhance resolution across the entire field of view, as demonstrated by the accumulated localized image (Figure 2h; Video S1, Supporting Information).

### 2.3 Multi‐Modal Volumetric Super‐Resolution Imaging of the Murine Brain

**Figure 3:**
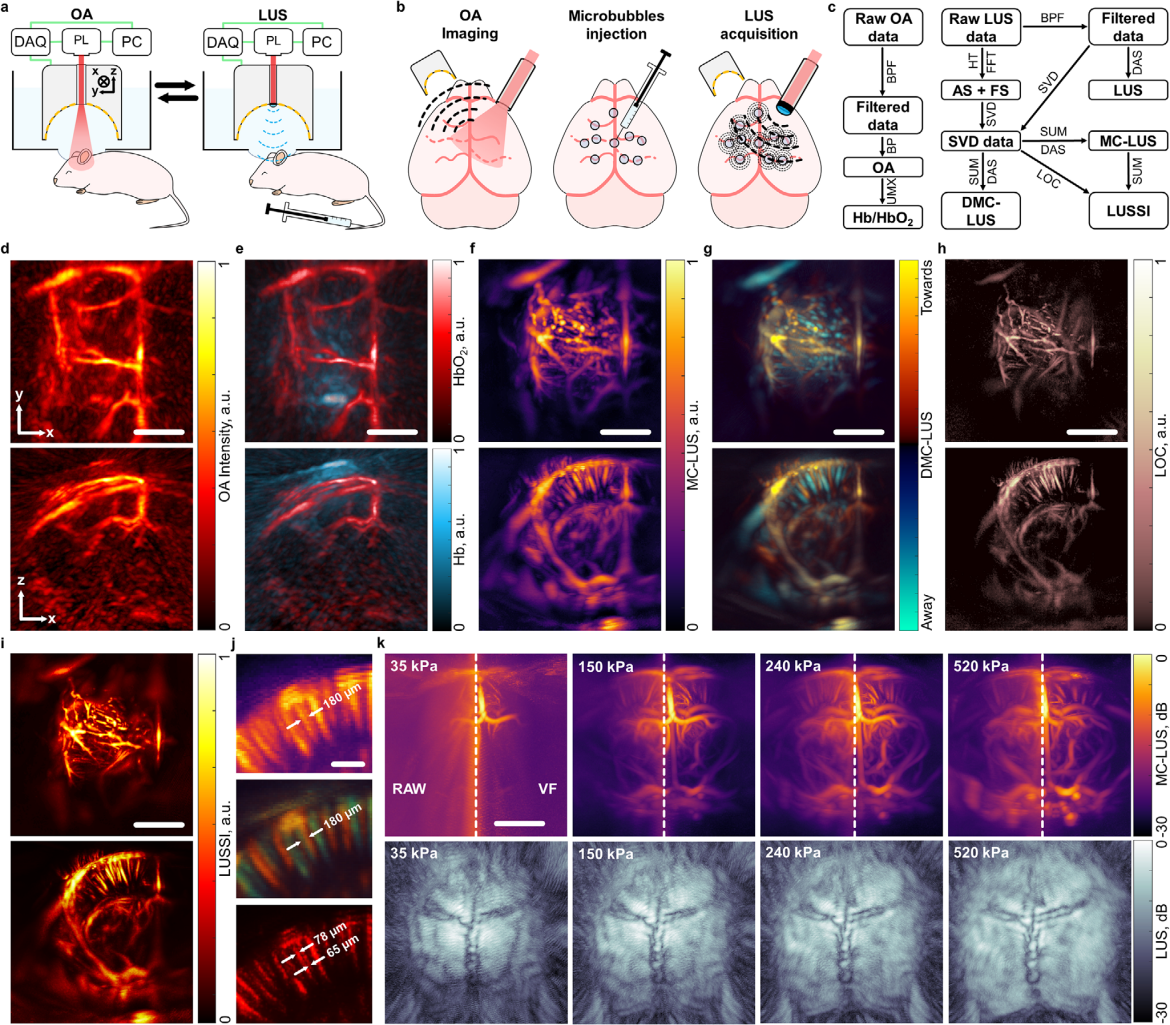
Multi‐modal optoacoustic (OA) and laser ultrasound (LUS) imaging of the murine brain. a) Experimental set‐up for the sequential acquisition of OA and LUS images: DAQ – multi‐channel data acquisition system, PL – tunable nanosecond pulsed laser, PC – personal computer. b) Schematic representation of OA and LUS responses in the brain. c) Schematic representation of the processing pipeline for OA and LUS images. BPF – bandpass filter, BP – back‐projection, UMX – unmixing, Hb/HbO_2_ – unmixed deoxy‐ and oxyhemoglobin components, HT – Hilbert transform, FFT – fast Fourier transform, AS – analytic signal, FS – frequency splitting, DMC‐LUS – directional motion contrast LUS, SVD – singular value decomposition filter, DAS – delay and sum, SUM – sum along the temporal dimension, MC‐LUS – motion‐contrast LUS, LOC – localization, LUSSI – laser ultrasound super‐resolution imaging. d) Maximum intensity projections (MIPs) of the 3D OA image at 800 nm. e) MIPs of the 3D bio‐distributions of oxygenated and deoxygenated hemoglobin unmixed from multi‐spectral (multi‐wavelength) data. f) MIPs of the 3D motion‐contrast LUS (MC‐LUS) image representing microbubbles flow in the blood. g) MIPs of the 3D directional motion‐contrast LUS (DMC‐LUS) image displaying two flow components, moving either toward the transducer or away from the transducer. h) MIPs of the 3D localization image are built by superimposing the localized positions of microbubbles. i) LUS super‐resolution imaging (LUSSI) is achieved by combining the MC‐LUS and localization images. j) Detailed views of pial microvasculature and penetrating arterioles and venules indicating the resolution achieved via localization of particles. k) MIPs of the original (left part) and Frangi (vesselness) – filtered (right part) MC‐LUS images (top) along with the LUS images (bottom) for different peak pressures of the incident US wave. Scalebars – 2 mm (d‐i and k) and 500 µm (j).

Super‐resolution US angiography and multi‐spectral OA imaging both rely on sensitive detection with piezoelectric array transducers. In super‐resolution US, microbubble contrast agents are tracked individually to visualize blood flow at a microscopic level, while in OA imaging, wavelength‐tunable lasers excite hemoglobin absorption to map oxygenation states. To integrate these modalities, a spherical array transducer was used with interchangeable illumination: a fiber bundle for OA excitation and a LUS emitter for super‐resolution US imaging (**Figure** **3**a). Time‐resolved US signals were acquired for both modalities (Figure S2a,b, Supporting Information), enabling multi‐modal volumetric imaging. Here, 3D LUS data could serve as an anatomical reference for other imaging modalities since it depicts the cranial sutures position and bregma. OA signals were generated using a tunable nanosecond laser, while contrast‐enhanced LUS was achieved following intravenous microbubble injection (Figure 3b). Dedicated processing pipelines were developed to optimize image reconstruction for both multi‐spectral OA and super‐resolution LUS imaging (Figure 3c; see Experimental Section for details).

LUS imaging of the same subject shows pronounced backscatter from the skull (Figure S2c, Supporting Information), but also indicates efficient transcranial US propagation, as reflected by strong signals from deeper regions. Motion‐contrast LUS (MC‐LUS) imaging visualizes blood flow across the mouse brain (Figure 3f), and directional motion‐contrast LUS (DMC‐LUS) additionally provides information on flow direction (Figure 3g). MC‐LUS images can be generated with relatively few frames, allowing fast imaging speeds. Indeed, vascular structures become clearly visible after 200 frames (2 s of acquisition, Figure S3, Supporting Information).

Further resolution enhancement is achieved by localizing individual microbubble signals and superimposing their positions, thereby overcoming the acoustic diffraction limit (Figure 3h). The final LUSSI image combines MC‐LUS and localization data, effectively leveraging both high‐contrast and super‐resolution information (Figure 3i; see Experimental Section). Detailed views of the pial microvasculature and penetrating vessels highlight the improved resolution achieved through localization, where the resolution of the typically‐resolvable structure reached 34 µm, which is ≈5× improvement with respect to the resolution, measured with conventional LUS (Figure 3j; Figure S4, Supporting Information). DMC‐LUS further reveals inhomogeneous flow direction within a structure that appears to represent a single vessel in the MC‐LUS image. The LUSSI image confirms that this apparent inhomogeneity is due to the presence of two closely spaced vessels. Rotating views of the reconstructed images further illustrate the multi‐parametric imaging capability of the approach (Video S2, Supporting Information).

An important consideration is the non‐linear dependence of microbubble response on the amplitude of the incident US beam (Figure 3k). At low excitation pressures (≈35 kPa, corresponding to 1.5 mJ per‐pulse optical energy), standard LUS images reveal cranial suture structures, but microbubble signals remain near the noise level. Increasing the pressure to 240 kPa (7.5 mJ per‐pulse) substantially improves microbubble contrast. Further pressure increases up to 520 kPa (14 mJ per‐pulse) enhance deep vessel visualization but reduce cortical vessel detection, attributed to inertial cavitation and destruction of microbubbles in cortical regions,^[^ ^69^ ^]^ thereby defining an optimal pressure range for imaging. Finally, applying a Frangi vesselness filter to MC‐LUS images improves vascular network visibility (Figure 3k), though the method's susceptibility to artifacts and its limited accuracy remain concerning.^[^ ^75^ ^]^

### 2.4 Angiographic Markers for Localization of Glioblastomas in the Brain

Imaging‐based methods are widely used to detect and monitor the progression of brain tumors, particularly glioblastomas. In this study, we employed LUSSI for volumetric, high‐resolution imaging of brain tumors in a preclinical glioblastoma model to capture vascular changes. These may facilitate longitudinal tumor detection and localization, which play a critical role in preclinical studies for validating the expected tumor growth and selecting the most effective time points for testing new therapeutic strategies. Since MRI and magnetic resonance angiography (MRA) in particular are considered the gold standard for brain tumor imaging, they were used to validate the vascular and anatomical features observed with LUSSI.

**Figure 4:**
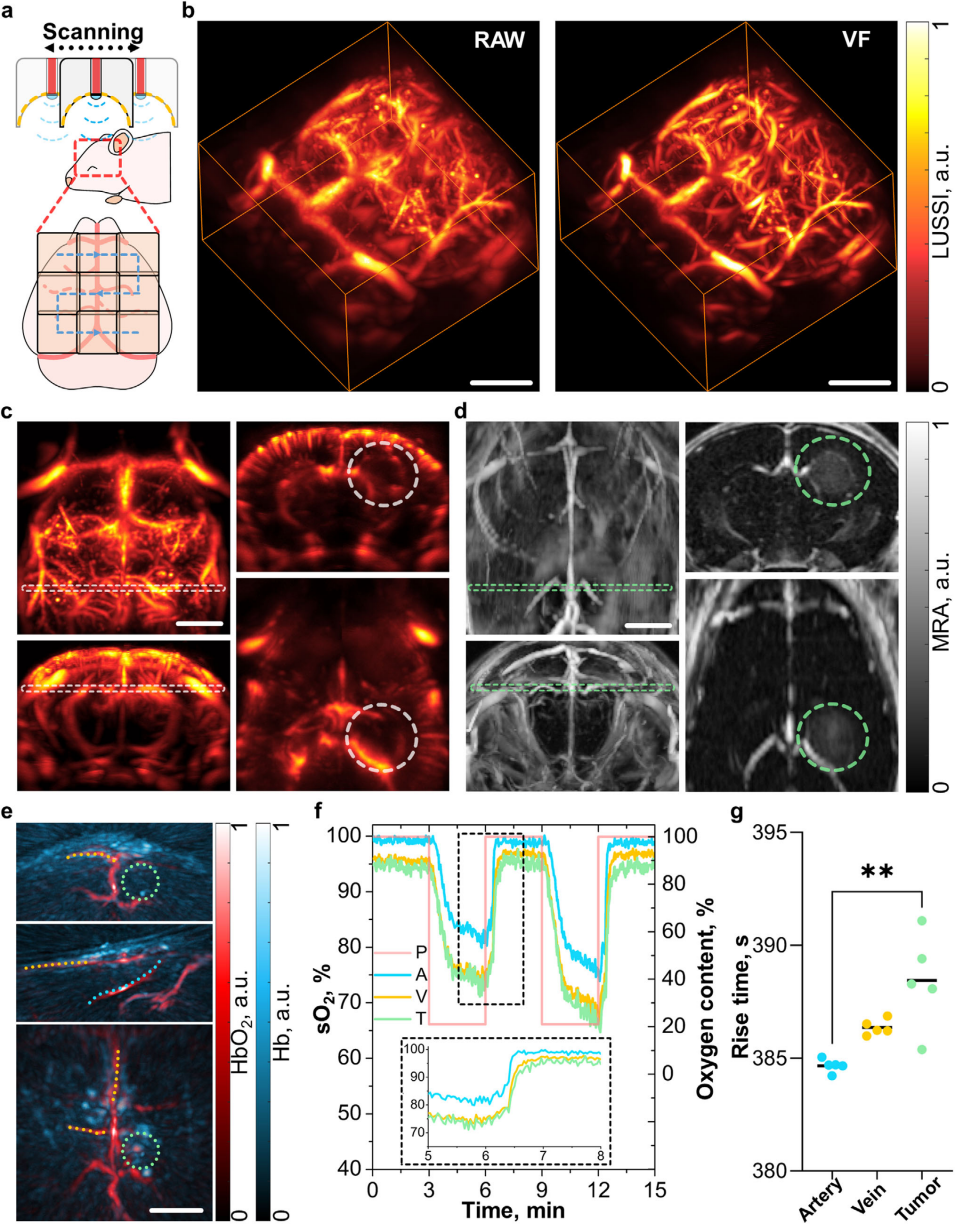
Whole‐brain tumor detection and localization with laser ultrasound (LUS), optoacoustic (OA), and magnetic resonance angiography (MRA). a) Schematic representation of the multi‐position acquisition setup. b) 3D views of the image reconstructed with laser ultrasound super‐resolution imaging (LUSSI) (left) and of the equivalent image after being processed with a Frangi vesselness filter (VF, right) for a mouse at day 17 post inoculation of U‐87 MG cells. c) Maximum intensity projections (MIPs) of the whole‐brain LUSSI image (left) along with selected coronal and transverse cross sections (right). The tumor location and selected sections are indicated with dashed rectangles and circles, respectively. d) MIPs of the whole‐brain MRA image (left) along with selected coronal and transverse cross sections (right). The tumor location and selected sections are indicated with dashed rectangles and circles, respectively. e) Bio‐distributions of oxygenated and deoxygenated hemoglobin unmixed from multi‐spectral (multi‐wavelength) OA data. f) The breathing gas challenge paradigm (P, pink) along with average temporal variations of the measured oxygen saturation values at 5 selected positions in an artery (A, blue), shallow and deep veins (V, yellow), and tumor vessels (T, green). The inset shows a zoom‐in of the oxygen saturation profiles during the change of breathing gas from air to pure oxygen. g) Rise time for the 5 selected points in an artery (dashed blue curve in (g)), shallow and deep veins (dashed orange curves in (g)), and tumor (dashed green circle in (g)) vessels; Kruskal‐Wallis test, followed by Dunn's multiple comparisons test, p = 0.0044. Scalebars – 2 mm.

LUS can cover the full depth of the brain till the circle of Willis for a single position of the array but has a limited FOV in the lateral directions. To improve the visibility of the peripheral regions of the murine brain, the array was then raster‐scanned at 9 positions with a 4‐mm step (**Figure** **4**a). By compounding (superimposing) the reconstructed images at each scanning position, LUSSI unveils vascular networks spanning the entirety of the brain (Figure 4b; Video S3, Supporting Information). A large area of the brain could also be covered by superimposing the images acquired at two positions corresponding to the left and right hemispheres (Figure S5, Supporting Information). The glioblastoma location and dimensions for a mouse, injected with U‐87 MG cells (Figure S6a, Supporting Information) 17 days before the experiment, can be established by examining specific sections of the 3D image (Figure 4c). Specifically, the tumor appears as a void‐like low‐contrast region surrounded by blood vessels (Video S4, Supporting Information), as expected when angiogenesis is produced around a necrotic core in response to hypoxic and nutrient‐deprived conditions.^[^ ^76^ ^]^ The compression of the corpus callosum vasculature because of the tumor position and the shortening/compression of the cortical penetrating vessels in the region above the tumor could be also noted. The gadolinium‐enhanced MRA image of the same mouse reveals identical vascular pattern, aligning with the tumor location identified in the MC‐LUS image (Figure 4d).^[^ ^77^, ^78^ ^]^ Gadolinium extravasation from the vasculature provides additionally contrast, enhancing visualization and delineation of the tumor mass. It could be noted that the LUSSI images in the presented conditions demonstrate more details in the penetrating cortical vessels in comparison to MRA, although the vessel detection ability becomes comparable when looking at the entire brain slice (Figure S7, Supporting Information).

Monitoring of tumor growth was also achieved by acquiring LUSSI images at different time points following inoculation of tumor cells to monitor tumor growth, validated by MRA (Figure S6, Supporting Information). Mice were scored during longitudinal experiments, with body weights and behavior being carefully monitored (Figure S6b, Supporting Information). The evidence of progression of vascular distortion and new vessel formation during the 7‐day period between imaging sessions (13 and 20 days following injection of cells) was captured (Figure S6c–f, Supporting Information).

The capability of OA to resolve oxygen saturation levels can also help in the identification of the tumorous regions. Low oxygenation was observed at the expected location of the tumor, consistent with hypoxic conditions (Figure 4g). The mouse was further subjected to a breathing gas challenge to evaluate dynamic patterns (Figure 4f, pure air to pure oxygen, oxygen content as the pink line in Figure 4f). Remarkably, a distinct time profile of oxygen saturation was detected within the tumor region, differing from those observed in the vein (Figure 4f,g; Figure S8, Supporting Information). This finding underscores the significance of dynamic OA imaging for the detection of tumors in the brain, for which a spherical array geometry is essential.

### 2.5 Assessment of Tumor Heterogeneities

**Figure 5:**
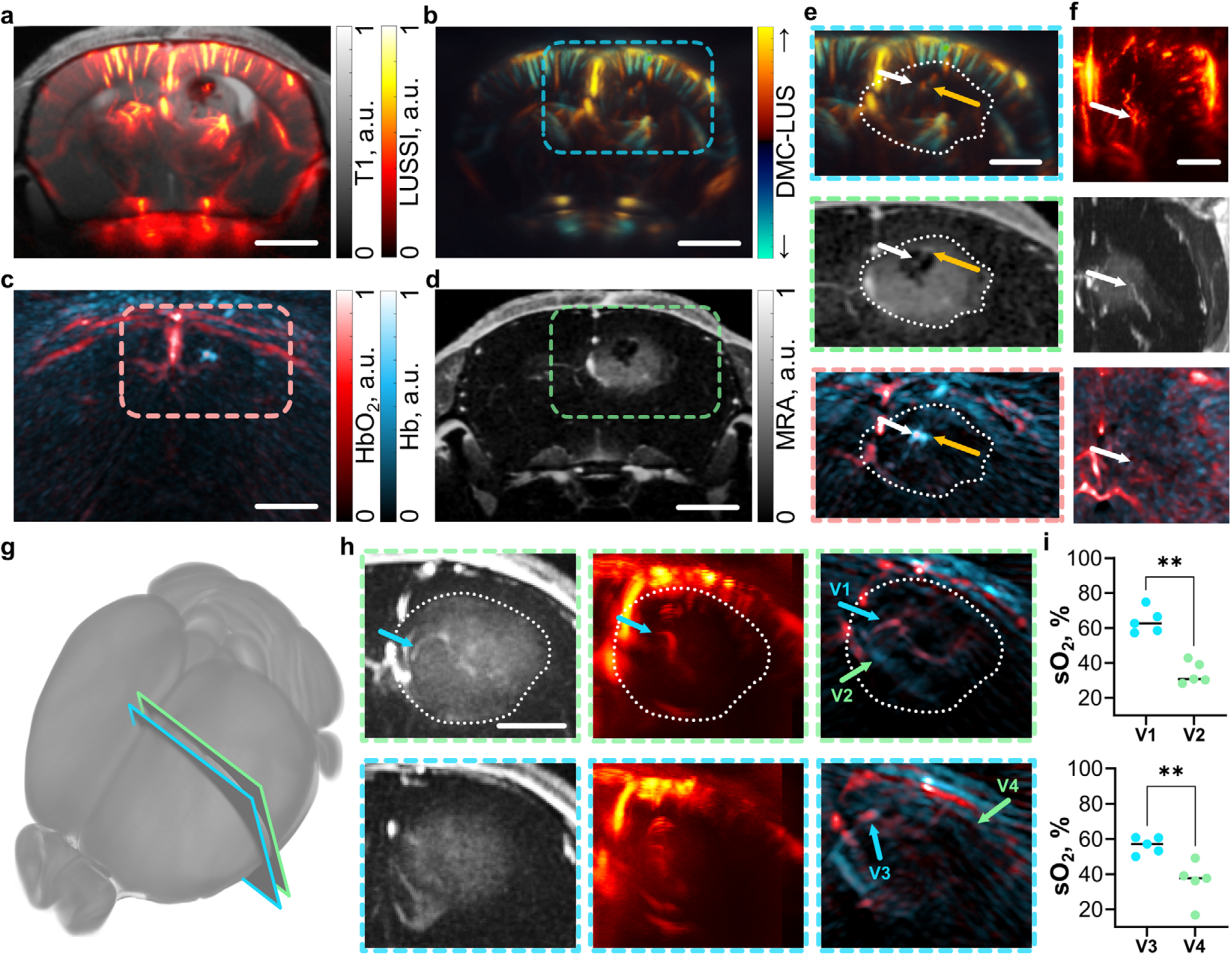
Visualization of tumor heterogeneities with laser ultrasound (LUS), optoacoustic (OA), and magnetic resonance angiography (MRA). a) Coronal section of the image of a mouse at day 36 post‐inoculation of U‐87 MG tumor cells rendered with laser ultrasound super‐resolution imaging (LUSSI) superimposed to the equivalent T1‐weighted MRI image. b) Equivalent coronal section of the directional motion‐contrast LUS (DMC‐LUS) image displaying flow direction. c) Equivalent coronal section of the multi‐spectrally unmixed OA image displays the bio‐distributions of oxygenated and deoxygenated hemoglobin. d) Equivalent coronal section of the magnetic resonance angiography (MRA) image. e) Zoom‐ins of the DMC‐LUS (top), MRA (middle), and OA (bottom) coronal sections for the regions indicated in (b), (c), and (d), respectively. White arrows indicate the vessel position, orange arrows indicate the internal hemorrhage of the tumor around the vessel. f) Transverse sections of the tumor area in LUSSI (top), MRA (middle), and OA (bottom) images. White arrows indicate the same vessel as in (f). g) Coronal sections indicating the tumor location in a mouse imaged at day 36 post‐inoculation of tumor cells. h) MRA (left), LUSSI (middle), and OA (right) images for the sections indicated in (g). The tumor volume is indicated with a dashed white line. i) Measured oxygen saturation values at selected vessels in the tumor indicated in (h). Mann‐Whitney test, p = 0.0079 for both pairs. Scalebars – 2 mm (a‐f), and 1 mm (h).

Tumor heterogeneity is a key factor driving the aggressive growth, invasive behavior, and variable treatment response of glioblastomas. Accurately assessing this heterogeneity is essential for understanding tumor progression and developing more effective therapies. The multi‐parametric imaging capabilities of LUSSI, enhanced with OA contrast, offer valuable insights into the tumor microenvironment that complement conventional MRI‐based approaches. As shown in **Figure** **5**, LUSSI provides angiographic contrast that reveals intra‐tumoral vascular features in late‐stage glioblastomas (36 days post U‐87 MG cell injection). One representative example shows a tumor region containing a central void‐like structure with no apparent US contrast. LUSSI highlights compressed vessels in the corpus callosum and cortex, delineating the tumor boundary (Figure 5a). Within the central void, a small, bright, point‐like structure is visible, perfused with microbubbles, suggesting its vascular nature. DMC‐LUS confirms this interpretation by showing alternating flow directions within the structure (Figure 5b). Multi‐spectral OA imaging further indicates elevated oxygenation at the center of the void, surrounded by a region of low oxygen saturation, consistent with a perfused vessel and adjacent hemorrhagic area (Figure 5c). MRA data also supports perfusion of the central structure but lacks functional information about the surrounding void (Figure 5d). Additional zoomed‐in views and alternate slice projections provide further evidence (Figure 5e,f). In particular, the coronal MRA section shows a hypointense region consistent with a hemorrhage (orange arrow, Figure 5e).

The complexity of tumor is more evident in two coronal sections of a larger glioblastoma (36 days post‐tumor cell injection). A prominent vessel with a convoluted path crossing the entire tumor was observed in MRA, LUSSI, and OA images (green arrow in Figure 5h). Vascular networks, potentially indicative of newly formed blood vessels, are also clearly visible inside and around the tumor, while distortion of major vascular structures is also evident. Oxygen saturation in this area is shown to exhibit pronounced heterogeneities. The relatively high oxygenation of the vessel traversing the tumor suggests that oxygen and nutrients are being supplied to internal regions, which may serve as an indicator of tumor aggressiveness. In contrast, other tumors have been shown to be characterized by low blood oxygenation at the core.^[^ ^79^, ^80^ ^]^ The capability to quantify tumor heterogeneities is better illustrated when analyzing blood oxygen saturation values at selected tumor vessels (arrows in Figure 5h). Significant differences in oxygen saturation for selected voxels in several vessels across the tumor volume, indicative of heterogeneous hypoxic profiles, were observed (Figure 5i). LUSSI enhanced with OA contrast can then shed new light on how hypoxia influences tumor growth, progression, and response to therapy.

## 3 Discussion and Conclusions

Super‐resolution US has greatly impacted biomedical research by enabling non‐invasive angiographic imaging at previously‐unavailable resolutions beyond the acoustic diffraction limit,^[^ ^81^, ^82^, ^83^, ^84^ ^]^ shedding new light on disease‐related vascular changes and therapeutic responses.^[^ ^85^, ^86^, ^87^, ^88^, ^89^ ^]^ As super‐resolution US relies on intravenous administration of FDA‐approved contrast agents, such as microbubbles, it is well‐positioned for clinical translation, with diagnostic potential spanning multiple areas including oncology, cardiology, and neurology.^[^ ^21^, ^90^, ^91^, ^92^ ^]^ Ongoing developments focus on performance enhancement, reduction of system complexity and cost, and integration with complementary imaging modalities. Spherical array transducers provide a practical solution for 3D localization of microbubble responses by capturing US responses across a wide angular aperture.^[^ ^61^, ^62^ ^]^ Similarly, OA imaging increasingly uses spherical arrays to achieve accurate tomographic reconstructions and extract quantitative molecular‐specific information.^[^ ^93^, ^94^, ^95^, ^96^ ^]^ The LUSSI platform proposed in this work integrates these capabilities by delivering coaxial US and light beams through the central aperture of a spherical array for efficient volumetric excitation of the same area. This approach was shown to enable generation of sufficiently strong US waves for localization of microbubble signals without the need for image compounding. Using the same aperture for light delivery also facilitates OA imaging in the same region, with a shared cost‐effective acquisition system operating in passive reception mode.

LUSSI's capabilities to assess tumor heterogeneities were shown in a murine glioblastoma model. Glioblastoma is the most aggressive primary brain tumor, with poor prognosis and high resistance to current treatments. Despite advances in surgery, radiation, and chemotherapy, median survival remains 15–23 months, and 5‐year survival is below 6%.^[^ ^97^ ^]^ Preclinical mouse models play a crucial role in studying tumor biology and evaluating early therapeutic interventions.^[^ ^98^ ^]^ A key challenge in glioblastoma management is its pronounced heterogeneity, which contributes to variable progression and treatment response. As a complex and multi‐faceted phenomenon, cancer heterogeneity requires new approaches for thorough characterization. Imaging techniques capable of assessing heterogeneities in brain tumors are essential for developing more effective and personalized therapies.^[^ ^99^ ^]^ While MRI provides excellent soft‐tissue contrast and volume segmentation, it often lacks sensitivity to treatment‐induced microstructural changes. Vascular features such as neovascularization, better captured by MRA, may correlate more closely with clinical outcomes.^[^ ^100^, ^101^, ^102^, ^103^ ^]^ Our results show that LUSSI, particularly when combined with OA imaging, can identify vascular distortions and hemodynamic heterogeneities and complement the powerful performance of MRA. For example, structural abnormalities in major vessels were clearly observed with LUSSI and corroborated by MRA. OA imaging added dynamic oxygenation measurements, revealing intratumoral variations in perfusion and hypoxia. In addition, combining contrast agents of different sizes, e.g., microbubbles for flow quantification and nanoparticles for permeability assessment, can further extend the imaging capabilities of this hybrid approach. Both super‐resolution US and OA imaging have already demonstrated feasibility for resolving vascular structures in the human brain, overcoming challenges such as skull‐induced attenuation and acoustic aberrations.^[^ ^19^, ^50^, ^55^ ^]^

The proficient performance of LUSSI for non‐invasive functional angiographic imaging of the murine brain also anticipates new discoveries in neuroscience, particularly if combined with OA imaging. Innovative imaging tools enabling new findings on the mouse brain play a crucial role in advancing our understanding of brain function. The preference for mouse models in neuroscience stems from their genetic affinity with humans, the availability of advanced genetic tools, and the capability to replicate human diseases.^[^ ^104^ ^]^ Microvascular alterations in the brain are known to occur in Alzheimer's disease, Parkinson's disease, and other neurodegenerative conditions,^[^ ^105^ ^]^ with available transgenic mouse strains recapitulating specific aspects of these conditions.^[^ ^106^ ^]^ On the other hand, high‐resolution angiographic imaging is essential to reveal the intricate structure and connectivity of the mammalian brain. Spontaneous or stimulus‐evoked brain activity can further be characterized with functional imaging techniques. In this regard, the unique capability of super‐resolution US imaging to quantify microvascular blood flow is essential to characterize hemodynamic changes associated to neural activity. OA imaging further enables quantification of additional functional parameters such as changes in oxygen saturation and in the concentrations of oxygenated, deoxygenated, and total hemoglobin.^[^ ^107^, ^108^, ^109^ ^]^ Taking together, hybridization of LUSSI with OA can provide unprecedented capabilities for multi‐parametric assessment of the brain in action, particularly considering that localization optoacoustic tomography (LOT) has further been developed as a super‐resolution OA imaging approach.^[^ ^39^, ^110^, ^111^, ^112^, ^113^, ^114^, ^115^, ^116^ ^]^

The LUSSI methodology is flexible and can be adapted for various implementations, depending on application‐specific requirements. A key component is the light‐absorbing layer that converts laser pulses into US waves. Low‐cost materials such as PDMS embedded with light‐absorbing nanoparticles, including carbon‐based materials, can be used to tailor the shape and amplitude of the generated US beam.^[^ ^27^, ^117^, ^118^, ^119^, ^120^ ^]^ PDMS‐candle soot composites have shown high optical‐to‐acoustic conversion efficiency and are easy to fabricate.^[^ ^121^ ^]^ Other designs based on absorbing polymers or carbon nanostructures have been proposed to further enhance pressure output.^[^ ^27^, ^122^, ^123^, ^124^ ^]^ In alternative implementations, a semi‐transparent emitter allows coaxial light and US delivery, enabling simultaneous acquisition of OA and LUS signals (OPLUS imaging).^[^ ^34^ ^]^ Miniaturization of LUS emitters—for example, integrating them onto optical fiber tips—opens possibilities for endoscopic or intravascular applications,^[^ ^32^, ^125^ ^]^ particularly in anatomically constrained regions. Note that unlike the non‐invasive approach proposed in this work, it is crucial to recognize that catheter‐based interventions are, by definition, minimally‐invasive procedures with corresponding clinical considerations.

LUSSI's frame rate and data density are constrained by the laser pulse repetition frequency (PRF). Optical parametric oscillator (OPO)‐based lasers, needed for multi‐spectral OA acquisitions, typically operate at PRFs up to 100 Hz. However, imaging rates can potentially be increased to the kilohertz range.^[^ ^126^ ^]^ Emerging light sources such as high‐pulse‐power diode lasers and LEDs offer cost‐effective, compact alternatives suitable for LUSSI, provided compatible absorbing materials are used.^[^ ^27^, ^28^, ^127^ ^]^ LUS emitter degradation over time and variability in output pressure are potential limitations. However, fabrication of LUS emitters is straightforward, and standardization of carbon coating and PDMS application can improve reproducibility. Automated fabrication systems may further enhance consistency. Tissue motion is also expected to result in image artefacts that hamper registration of high‐resolution US and OA images. Motion estimation and correction in localization‐based angiography can be achieved, e.g., with the SVD‐filtered image sequence corresponding to the tissue background.^[^ ^128^, ^129^ ^]^ Tissue heterogeneities, particularly in the presence of heterogeneous tumor structures, may also result in image artefacts and registration inaccuracies. Advanced algorithms, e.g., accounting for differences in speed of sound between different tissues and the water coupling medium may them be needed for optimal performance.^[^ ^46^ ^]^ Another aspect that can affect LUSSI's performance is the relatively short circulation time and variability in concentration of intravenously‐injected microbubbles. Alternative contrast agents with prolonged circulation or controlled continuous injections may help address this limitation.^[^ ^53^, ^81^ ^]^

The clinical translation of LUSSI is supported by the fact that all the underlying materials and technologies are FDA‐approved or licensed for human use. Although the human skull is significantly thicker and induces much stronger acoustic aberrations than the murine cranial bones, transcranial super‐resolution US imaging through the natural temporal bone window has been successfully demonstrated,^[^ ^19^ ^]^ suggesting a potential route for LUSSI application. Similarly, the feasibility of transcranial OA imaging has also been demonstrated, which is facilitated by the unidirectional propagation of US waves.^[^ ^130^, ^131^ ^]^ The demonstrated laser‐induced pressures ≈500 kPa can be increased by optimizing the emitter geometry or laser energy for more efficient transcranial delivery. Additionally, the insulating PDMS layer in the LUS emitter prevents direct contact between absorbing materials and the skin, allowing, e.g., the use of stronger absorbers (e.g., quantum dots, carbon nanostructures) with minimal biocompatibility concerns.

In conclusion, LUSSI represents a novel, non‐invasive methodology for high‐resolution exploration of microvascular structures with a demonstrated proficient performance in angiographic imaging of the mouse brain and characterization of heterogeneities in glioblastomas. By employing laser generation of US and spherical‐array detection of microbubble responses, LUSSI can break through the acoustic diffraction barrier and facilitate the rendering of the 3D volumetric vascular maps with further enriched OA functional contrast for maximizing the amount of information provided. This unique capability is expected to pave the way for groundbreaking insights into the microvascular changes associated with various diseases. The demonstrated good performance of the hybrid approach for multi‐parametric characterization of glioblastomas opens up new avenues for brain cancer research, paving the way for personalized treatments and long‐term tracking of therapeutic responses.

## 4 Experimental Section

### Materials

Sylgard 184 PDMS base and curing agent were sourced from Distrelec Schweiz AG, natural beeswax candles were obtained from Migros. NBK7 glass rods, 4 mm in diameter and 45 mm in length, were supplied by Nanyang Jingying Trade Co. Ltd. The Sonovue ultrasound contrast agent was acquired from Bracco, and 90 µm polystyrene spheres were provided by Cospheric LLC. Additional materials, including low melting point agar, tannic acid, 20% Intralipid solution, Eagle's Minimum Essential Medium (EMEM), Fetal Bovine Serum (FBS), and Trypsin‐EDTA solution were procured from Sigma Aldrich. U‐87 MG glioblastoma line was purchased from ATCC. Hematoxylin and Eosin staining (H&E Staining Kit, ab245880) was purchased from Abcam.

### Laser Ultrasound Emitter Manufacturing

A 4 mm NBK7 glass rod was first cleaned in an ultrasonic bath using acetone and isopropanol for 15 min to remove any organic residues from the surface. One of the flat ends of the rod was then roughened with 200 grit SiC sandpaper and thoroughly rinsed to eliminate glass dust. Next, 10 mL of a standard PDMS blend (10:1 base to curing agent ratio) was mixed and degassed for 30 min in a vacuum chamber. The glass rod was positioned vertically, and 2 µL of the degassed PDMS was applied to the top and carefully spread to form a layer ≈150 µm thick. After curing the PDMS in an oven at 120 °C for 2 h (FD 23, BINDER GmbH), the emitter billet was removed and allowed to cool at room temperature for 15 min. Candle soot deposition was carried out using a modified commercial Cartesian‐grid 3D printer equipped with a custom‐built holder for glass rods (Figure S1, Supporting Information). The candle was placed on the printer's platform, and the distance from the wick tip to the PDMS surface was adjusted to 1 cm. After that the candle was lit, letting the flame to be stabilized for 1 min, and then the rod holder was lowered into the flame for 30s. The printer head was programmed to slowly move along the X‐axis 2 cm each side to prevent PDMS overheating and to enhance coating uniformity. The platform motion along the Y‐axis was avoided to prevent the disturbance of the flame. Following this, the carbon soot‐coated emitter was covered with an additional 1 mm layer of PDMS forming a dome. The rod was placed back in the oven for another 2 h to allow the PDMS to fully cure. Finally, the resulting Glass‐PDMS‐CS‐PDMS emitter was inserted into a 3D‐printed housing. The geometrical dimensions of the emitter were selected according to the technical specifications of the spherical array transducer used in this study to detect the ultrasound signal from the tissue (5 mm central aperture opening, 30 mm radius of curvature). PDMS as a coating material was chosen due to the almost perfect matching to water (typical impedance of 1.1 MRayl versus 1.48 MRayl for water) and its transparency to the NIR light.^[^ ^132^, ^133^ ^]^ The overall structure of the emitter was examined using scanning electron microscopy (SEM). The prepared LUS‐emitting tip was detached from the glass rod with a scalpel, immersed in liquid nitrogen for 10 s, and sectioned sagittally with a sharp blade.^134^ The specimen was mounted on carbon tape, fixed to the SEM stub, and sputter‐coated with a 2 nm Pt/Pd (80/20) layer using a CCU‐010HV (Safematic) coater to minimize charging. Imaging was carried out on a SU5000 SEM (Hitachi) operated at 3 kV in secondary electron (SE) mode. The resulting cross‐sectional micrographs revealed an amorphous carbon layer with spherical inclusions and an average thickness of ≈1.4 µm (Figure S1, Supporting Information).

### Laser Ultrasound Emitter Characterization

The pressure wavefield generated by the LUS emitter was measured using a 1 mm diameter PVDF needle hydrophone sensor (Precision Acoustics Ltd). The experiment was conducted in a thermally stabilized water tank preheated to 30 °C filled with deionized water. The emitter was aligned with the hydrophone, which was mounted on a 3‐axis motorized automated stage (IAI Inc.), and coupled to a 5 mm output silica optical fiber bundle. An OPO pulsed laser (SpitLight 1200, Innolas GmbH), with pulse width of 7 ns, repetition rate of 10 Hz, and wavelength tuned to 720 nm, was used to excite US signals in the carbon layer of the emitter by means of the OA effect. The pressure signal at 30 mm from the surface of the emitter was captured by a 12‐bit single‐channel waveform digitizer (ATS9351, Alazar Technologies Inc.) operating at a rate of 100 MSamp/s triggered by the laser. To assess the pressure distribution perpendicular to the US beam propagation axis, the hydrophone was scanned in a raster pattern, covering an area of 25 × 25 mm^2^ with uniform 1 mm steps along the x and y axes. The distance of 30 mm resembles the focal distance (radius) of the spherical array transducer that was used in the study, and 40 mm represents a typical imaging depth of 10 mm away from the focus for the further phantom and in‐vivo experiments. The axial pressure distribution was examined by collecting data along a horizontal slice of 25 × 40 mm^2^, spanning distances from 15 to 55 mm from the emitter's surface.

### Image Reconstruction and Processing

*Optoacoustic image reconstruction*: OA reconstruction was performed with a graphics processing unit (GPU)‐based back‐projection algorithm. A volume of interest (VOI) corresponding to a Cartesian grid of points with 25 µm voxel size was considered for reconstruction, where the geometrical parameters of the VOI were adapted for each experiment (parameters such as reconstruction centre and the reconstruction volume size in mm^3^). The reconstructed OA image at the *i*‐th voxel of the grid (*r_i_*) was calculated as

(1)

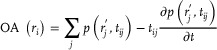

where $t_{ij}=\left|r_{i}-r_{j}^{\prime }\right|\;/c$ the time‐of‐flight between the *i*‐th voxel of the grid and the location of the *j*‐th element of the spherical array ($r_{j}^{\prime }$). Before reconstruction, the signals were band‐pass filtered between 0.2 and 8.5 MHz.

*Multi‐spectral unmixing*: For OA multi‐spectral unmixing, the acquired signals corresponding to different excitation wavelengths were first normalized with the laser energy transmitted through the fiber bundle and water tank measured with a pyroelectric sensor (Coherent, Inc.) in the range of 700 to 900 nm with step size of 10 nm. Images for different wavelengths were then reconstructed as described above and normalized with the estimated wavelength‐dependent optical fluence distribution. The fluence was estimated considering an exponential decay with depth (1D diffusion approximation), with exponential decay constant given by the effective attenuation coefficient 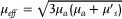, being μ_*a*_ and μ′_*s*_ the absorption and reduced scattering coefficients, respectively, approximated as uniform in the entire volume. The reduced scattering coefficient was taken from the literature for brain tissue.^[^ ^135^ ^]^ The absorption coefficient was considered to be proportional to the average OA intensity in the brain, with a value of 0.2 cm^−1^ at 800 nm (isosbestic point of hemoglobin) corresponding to an average of 5% v/v concentration of blood in tissue. The bio‐distributions of oxyhemoglobin and deoxyhemoglobin were unmixed via linear spectral fitting of the normalized images to the corresponding spectra. The absorption spectra for the two forms of hemoglobin were obtained from the literature.^[^ ^136^ ^]^

### Laser Ultrasound Image Reconstruction

LUS reconstruction was performed using a delay‐and‐sum approach implemented on a GPU. This method is analogous to back‐projection reconstruction but accounts for both the forward and backward propagation of US waves when calculating the times of flight. In the typical experiment, the emitter's position was first calibrated by imaging a single‐sphere phantom located at the acoustic focus of the transducer. Based on that, an offset insertion for the fiber bundle was modeled and 3D‐printed to position the emitter at the same position (30 mm from the focus) for all the experiments. A grid of the same size as for OA reconstruction was considered. The reconstructed LUS signal at the position of the *i*‐th voxel of the grid (*r_i_*) was calculated as

(2)

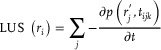

where 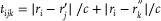 is the time‐of‐flight between the *i*‐th voxel of the grid and the location of the *j*‐th element of the spherical array ($r_{j}^{\prime }$) plus the time‐of‐flight between the position of the tip of the emitter (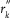) and the *i*‐th voxel of the grid. The signals were band‐pass filtered between 1 and 8.5 MHz before reconstruction. A Frangi vesselness filter was applied to some of the images to enhance vessel contrast, but only the unfiltered images were used in any further data analysis.

### Motion Contrast Laser Ultrasound Imaging

The acquired LUS datasets, collected at 24 MSamp/s sampling rate, consist of 3 dimensions: *N_s_* × *N_el_* × *N_tp_*, where *N_s_* is the number of collected samples (496 samples), *N_el_* is the number of elements of the spherical array (512 elements), and *N_tp_* is the amount of collected frames (typically 5 min, corresponding to 30 000 frames at 100 Hz frame rate). First, the raw data was spatially pre‐filtered in a range from 1 to 8.5 MHz using a Butterworth‐like filter and then arranged into the subsets of (496 × 512 × 400) size. Each subset was then cleaned from the tissue clutter using an SVD filter, with the first 100 eigenvectors removed. The filtered subsets were then reconstructed using a delay‐and‐sum approach code running on a GPU, and then the maximums of each 3D frame were stacked together and summed up to form MC‐LUS image. If several positions were acquired, the data was stitched and overlaid to show the full view of the brain.

### Directional Motion Contrast Laser Ultrasound Imaging

The acquired LUS datasets corresponding to time points after microbubble injection were further processed to extract bi‐directional information on the blood flow. For this, we modified an algorithm taken from the literature.^[^ ^48^ ^]^ First, the dataset was split into subsets of (496 samples × 512 elements × 400 frames). For each subset, the Hilbert transform was applied to all the signal frames (496 samples × 512 elements) along the sample direction to obtain a complex analytic signal *AS*  =  *S* + *iHT*(*S*), where *S* is an initial signal (496 samples long) and *HT*(*S*) is the Hilbert transform of the initial signal *S*. Then, the Fast Fourier Transform (FFT) was performed on the resulting signal, shifting the zero frequency components to the center of the spectrum. The frequency axis $\left[\frac{-f_{sampling}}{2},\frac{f_{sampling}}{2}\right]$ of the FFT signal is shaped according to the sampling frequency, given by the laser repetition rate in the case of LUS (100 Hz). The signal was split into two components according to the positive $F_{+}=\left[\frac{-f_{sampling}}{2},0\right)$ and negative $F_{-}=\left(0,\frac{f_{sampling}}{2}\right]$ frequencies, corresponding to flow toward and away from the transducer elements. Split parts were then shifted and transformed back into the time domain via inversed FFT, resulting in two signal matrices. These were then SVD filtered with the above‐mentioned parameters, reconstructed into volumes, and summed up to obtain two MC‐LUS images representing blood flow toward and away from the array. The images were then overlaid to form the DMC‐LUS image.

### Laser Ultrasound Super‐Resolution Imaging

LUSSI images were obtained by combining MC‐LUS and localization images. After the subsets were SVD‐filtered, leaving only the signals of the moving microbubbles and reconstructed into a 4D‐volumes [200 × 200 × 200 pix] × 400 frames, localization of microbubbles was performed. In each reconstructed frame, local intensity maxima were detected, and small regions around these maxima were correlated to a measured PSF of the imaging system, extracted from the single‐sphere phantom data. The maxima with correlation coefficients above 0.3 were considered as bubbles. Localization of these bubbles was then further refined using a local quadratic fitting of the intensity maxima, and their positions were stored. To form a LUSSI image, the intensities and their corresponding positions were plotted on a 3D grid with the step of 10 µm.

### Image Stitching

Large‐scale mouse brain imaging was performed by combining multiple positions of the spherical array transducer. Full brain data were acquired using a step‐and‐go Cartesian scan with a 2 mm step size. Specifically, the collected raw OA and LUS data corresponded to 496 samples × 512 elements × 3 longitudinal positions × 3 transversal positions. Both LUS and OA 3D volumes were reconstructed and translated to the corresponding scan positions using the MATLAB function *imtranslate*. Finally, max compounding was performed to create a complete image. The same algorithm was used to image both the left and right hemispheres, but only for two positions.

All datasets were reconstructed and analyzed using a workstation equipped with a Core i7‐10700F 2.8 GHz CPU (Intel Inc.), 128 GB of DDR4 2933 MHz RAM, and a Nvidia Titan V GPU with 12 GB VRAM (NVIDIA Inc.).

### Statistical Analysis

Statistical analysis was performed in Prism Graphpad 10.0.3. To compare the rise and fall times for the arterial, venous, and tumorous regions, 5 points were selected in each region by an unbiased third person, forming 3 groups of 5 points in each dataset. A non‐parametric Kruskal‐Wallis test was used to compare all 3 groups followed by Dunn's multiple comparisons test.

Another person selected 5 points along 4 vessels in 2 slices of the OA image of the tumor. The data was then formed into 2 groups related to each of the slices, 2 vessels per slice, and 5 points per vessel. The oxygenation values in these points were then calculated and compared within the slice (2 separate datasets, 2 vessels in each) using a Mann‐Whitney test.

### Animal Experiments

*Animal models*: This study was performed following the Swiss Federal Act on Animal Protection and was approved by the Cantonal Veterinary Office Zurich (license #ZH182/2023). A total of 7 Athymic nude mice (Foxn1nu, Charles River Laboratories, USA, 8–13 weeks old) were imaged in this study. The animals were housed in individually ventilated, temperature‐controlled cages under a 12‐h dark/light cycle. Pelleted food (3437PXL15, CARGILL) and water were provided ad libitum.

*Intracranial injection of U‐87 MG cells*: Intracerebral tumor cell injection was performed in Athymic nude mice (Foxn1nu, Charles River Laboratories, USA, 6–10 weeks old, females = 3, males = 3). Before injection, the U87‐MG cells (Figure S6, Supporting Information) were cultivated in an incubator at 5% CO2, 95% Air, and 37 °C. The cells were grown in EMEM supplemented with 10% FBS, detached from the 75 cm^2^ cell flask using Trypsin‐EDTA solution, rinsed, and resuspended in PBS following the standard protocols of the manufacturer. The animal was first carefully placed into a transparent induction chamber, and the gas flow was set to 250 mL min^−1^ Oxygen and 500 mL min^−1^ Air. The isoflurane vaporizer was set to 5% for anesthesia induction and waited for 2–4 min until the animal's respiration rate was reduced to reach a stable status (60–70/min). Then the animal was transferred to the stereotactic frame while the mice were anesthetised with 1.75–2.5% isoflurane via a nose cone to maintain the anesthesia. During the U87‐MG cell intracranial injection procedure, an ophthalmic ointment (Lacrinorm, Bausch & Lomb) was applied to prevent eye drying out of the cornea, and body temperature was monitored with a rectal probe and kept at 36.5 °C (PhysioSuite, Kent Scientific). An incision was made on the scalp, and the skull was exposed. A burr hole was drilled into the skull (Bregma ‐1.5–1.8 mm, right lateral 0.8 mm (cortex) and 1.4 mm (hippocampus), depth 0.7 mm (cortex) and 1.5 mm (hippocampus)) by an automatic drill (Ideal Micro Drill Surgical Drill, Harvard Apparatus). A volume of 2 µL of PBS, containing 0.5–1 × 10^6^ U‐87 MG cells, was injected using a 10 µL syringe (NanoFil 10 µL Syringe, World Precision Instrument) and 33‐gauge bevelled needle (NF33BV, World Precision Instrument). The injection rate (200 nL min^−1^) was controlled by an infusion pump. After injection, the needle was left in place for ≈5 min before being slowly withdrawn. The craniotomies were sealed with bone wax, and the skin around the wound was sutured and glued with tissue glue. Mice were provided ad libitum with water containing the analgesic buprenorphine for three consecutive days to relieve postoperative pain after injection. The body weights of animals were measured for three consecutive days post‐injection and afterward at least three times a week until the terminal experiment.

*Immunohistochemistry staining of GBM orthotopic tumors*: To verify the tumor location, post‐imaging perfused mouse brains were fixed overnight in 4% paraformaldehyde and then equilibrated in 15% and 30% sucrose in 0.1 m PBS at 4 °C. 30 µm‐thick coronal sections were sliced using a cryotome (CM3050S, Leica). Free‐floating sections were washed in PBS, mounted on microscope slides, and incubated with Hematoxylin and Eosin staining (H&E Staining Kit, ab245880, Abcam). Wide‐field images were acquired using a microscope equipped with 2.5× NA 0.075 and 10× NA 0.45 objectives (Axioplan 2, Zeiss) for assessment of H&E staining. Digital images were minimally processed using ImageJ to enhance brightness and contrast.

### Imaging Experiments

*Phantom imaging*: Preliminary tests with a phantom were conducted to evaluate the imaging capabilities of LUS. First, 5 mg of 90 µm black polyethylene spheres were added to 5 mL of a 4 mg mL^−1^ aqueous tannic acid solution, and a tip sonicator (Q125, Qsonica) was used to achieve uniform distribution of the spheres. Next, a 1.3% aqueous agar solution containing 2% Intralipid was prepared, and 1 mL of the sphere‐containing solution was added under vigorous stirring. This mixture was then poured into a top‐cut 20 mL syringe and cooled to 4 °C to facilitate agar gelation. The resulting cylindrical phantom was placed in a thermally‐stabilized water tank, where it was co‐aligned with a 512‐element piezoelectric composite spherical array transducer with a central frequency of 10 MHz and >80% detection bandwidth (≈100% at −6 dB detection bandwidth, 140° or 1.3π solid angle coverage, Imasonic SaS). Images. Reference OA data were initially collected at an excitation wavelength of 800 nm to confirm accurate positioning. The LUS emitter, along with the light‐guiding fiber bundle, was inserted into the central aperture of the transducer array. Images were then collected at 720 nm excitation wavelength, a repetition rate of 10 Hz, and averaged over 1000 laser pulses. All the data acquisition was performed using a 512‐channel system with a digitizing frequency of 24 MSamp/s and 496 samples per channel at 40 dB amplification (Falkenstein Mikrosysteme GmbH).

*In vivo imaging of the mouse brain vasculature*: A healthy female athymic nude mouse (≈24 g body weight, 8 weeks old) was used for testing the LUS imaging performance in vivo. The mouse was placed on a heating pad to maintain a constant body temperature (37 °C) with the head fixed with a stereotactic frame. Body parameters (heart rate, breathing rate, and blood oxygenation) were monitored using PhysioSuite (Kent Scientific Corp.). The 512‐element piezoelectric composite spherical array transducer mentioned above was used to collect the echo signal from the animal tissue. The transducer was mounted on a 3‐axis motorized stage inside a portable water tank with a round aperture at its bottom side sealed with a polyethylene film. A 100 Hz optical parametric oscillator (OPO) laser (SpitLight EVO‐II, Innolas, Germany) with a pulse duration of ≈7 ns was used as an OA excitation source. The per‐pulse laser energy was set to ≈7 mJ at 720 nm excitation wavelength. First, the OA multispectral data in a range from 700 to 900 nm wavelengths was collected for further brain oxygenation study and for accurate positioning purposes. Then, the LUS emitter was inserted in the central aperture of the transducer, and a 100 µL of freshly prepared stock solution of Sonovue microbubbles was injected into the tail vein in 20 µl portions each minute during the scan for 5 min. To image 2 hemispheres, the transducer was translated in 2 positions with a step of 2 mm across the skull. To image the entire brain, the signals were collected during a 3 × 3 positions raster scan with uniform 2 mm steps along X and Y and 5 min acquisition per position (30 000 frames) to ensure the whole brain coverage. To assess the nonlinear effects to US amplitude, the laser energy was set to 1.5, 3.5, 7.5, and 14 mJ pulse^−1^, which corresponds to 35, 150, 240, and 520 kPa generated US pressure, respectively. The mouse brain exposed at these pressure values was imaged at the central position. After the experiment, a phantom consisting of 1% agar and 90 µm polystyrene spheres was imaged in one position to calibrate the distance between the LUS emitter and the spherical array transducer focus. The speed of sound was calibrated by scanning the same phantom with OA tomography shortly after the US scan.

*In vivo imaging of the orthotopic glioblastoma in mice*: Orthotopic tumor‐bearing mice were imaged at 13–36 days post transcranial injection of U87‐MG cells as described above. OA and LUS imaging at two positions corresponding to the two brain hemispheres (tumor and control sites) was performed as described above. OA data was collected at a wavelength range of 700–900 nm, and LUS data was collected for 5 min for each hemisphere following injection of microbubbles. MRI images were acquired in a 7T small animal scanner (Biospec 70/16, Bruker BioSpin MRI) equipped with a cryogenic quadrature surface coil (Bruker BioSpin AG). Common standard adjustments including calibration of the basic frequency, reference frequency power, and receiving gain were performed in ParaVision 6.0.1 MRI software suite. MRA images were acquired using a FLASH sequence with 15.6 × 15.6 mm^2^ FOV resampling 312 × 312 pix^2^ matrix (50 µm in‐plane resolution). In total, 80 slices in the anterior‐posterior direction were acquired with a slice thickness of 300 µm, repetition time (TR) of 12 ms, echo time (TE) of 2.285 ms, and 4 averages. Subsequently, a T1‐weighted scan with the same geometry was acquired as an anatomical reference for the MRA data using a T1‐FLASH sequence with TR = 1270.96 ms, TE = 3 .78 ms, and averaged over three times. All MRI data analysis and graphical representations were implemented in MATLAB software (Mathworks Inc.) and Analysis of Functional NeuroImages software (AFNI, NIH). MC‐LUS, OA, MRI, and MRA images were manually co‐registered by matching the major vessels visible in all the imaging modalities, and tumor volumes were segmented.

## Conflict of Interest

The authors declare no conflict of interest.

## Supporting information



Supporting Information

Supplemental Video 1

Supplemental Video 2

Supplemental Video 3

Supplemental Video 4

## Data Availability

The data that support the findings of this study are available from the corresponding author upon reasonable request.
