# Supplementary material for: Laser Ultrasound Super‐Resolution Imaging for Multi‐Parametric Non‐Invasive Volumetric Characterization of Brain Cancer
Source: Adv Sci (Weinh). 2025 Sep 30;12(47):e02298. doi: 10.1002/advs.202502298 (PMC12713108; doi:10.1002/advs.202502298)
Supplement: Supplementary file 1 — Supporting Information [file ADVS-12-e02298-s002.docx]

**Supplementary Information**

**Laser ultrasound super-resolution imaging for multi-parametric non-invasive volumetric characterization of brain cancer**

Daniil Nozdriukhin^1,2^, Yi Chen^1,2^, Shuxin Lyu^1,2,3^, Daniel Razansky^1,2^, Xosé Luís Deán-Ben^1,2^

^1^Institute for Biomedical Engineering and Institute of Pharmacology and Toxicology, Faculty of Medicine, University of Zürich, Winterthurerstrasse 190, Zurich, 8057 Switzerland

^2^Institute for Biomedical Engineering, Department of Information Technology and Electrical Engineering, ETH Zürich, Rämistrasse 101, Zurich, 8093 Switzerland

^3^Institute of Medical Technology, Shanxi Medical University, Taiyuan, 030001 China

**Supplementary Figures**

**
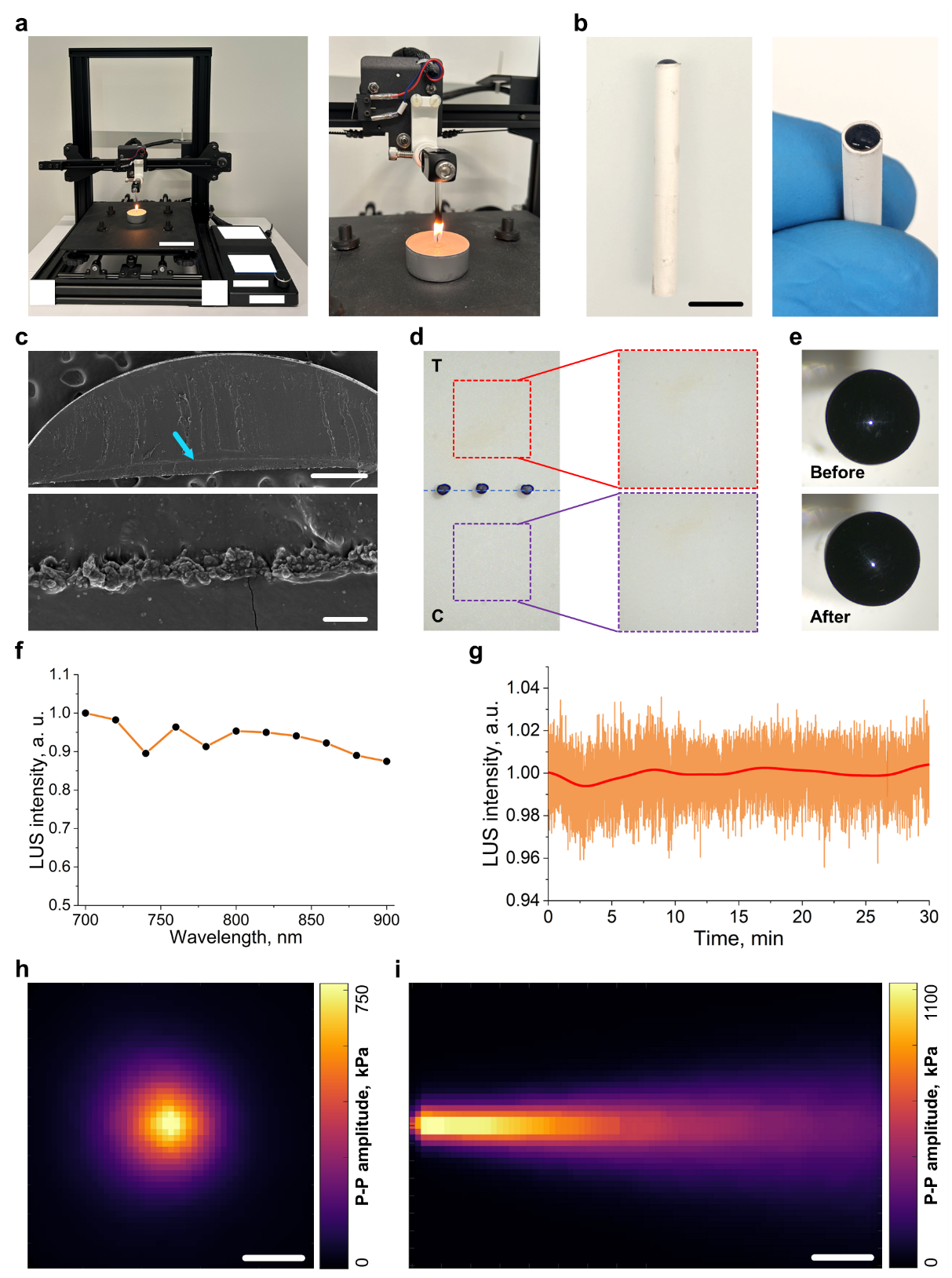
**

**Figure S1.** a) Photographs of the candle soot deposition setup**.** b) Photographs of the laser ultrasound (LUS) emitter. Scalebar – 1 cm. c) SEM microphotographs of the LUS emitter structure, demonstrating the interlayer of carbon between PDMS. Top image – overall view of the emitting dome (scalebar – 500 µm). An arrow points to the separation line between two layers of PDMS, where the carbon is deposited. Bottom image - zoom-in of the top image (scalebar – 2 µm ) with the visible deposited carbon structure. d) Photograph of a thin PDMS layer, applied between two glass slides. The top part (T) was subjected to 20000 laser pulses at 720 nm 16 mJ/cm^2^, while the bottom part below the dotted line was covered with a dark cloth and served as a control. The zoom comparison show no difference and no visible laser damage. e) Photographs of the LUS emitter before and after 20000 pulses at 720 nm, 16 mJ/cm^2^ laser fluence. f) LUS signal dependence on the excitation wavelength, acquired by pulsing the emiter to the submerged thick copper slab and acquiring the reflected signal. g) LUS stability plot, acquired by pulsing the emiter to the submerged thick copper slab and acquiring the reflected signal. h) Example of the ultrasound field generated by a 7 mm LUS emitter manufactured with the same method as the one used for the experiments at 30 mm distance perpendicular to the emitter axis. i) Example of the ultrasound field generated by this 7 mm LUS emitter at a plane parallel to the emitter axis covering a distance of 15-55 mm. It should be noted that while the overall amplitude is higher, the beam appears more narrow, tentatively because of the diffraction-based focusing effect. Scalebar – 5 mm.

**
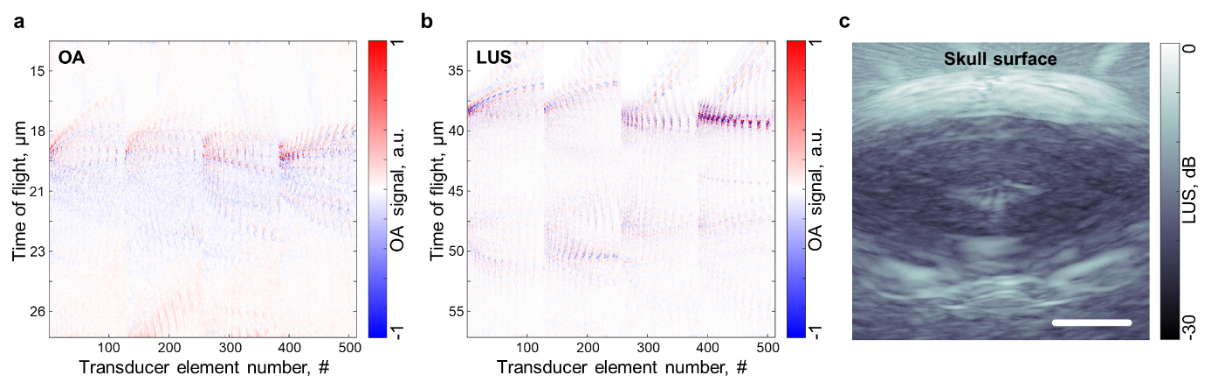
**

**Figure S2.** a) Typical OA signals (columns) from the mouse head acquired with the 512-element spherical array. b) Typical LUS signals (columns) from the mouse head acquired with the 512-element spherical array. c) LUS image of the mouse head in dB scale. Scalebar – 2 mm.

**
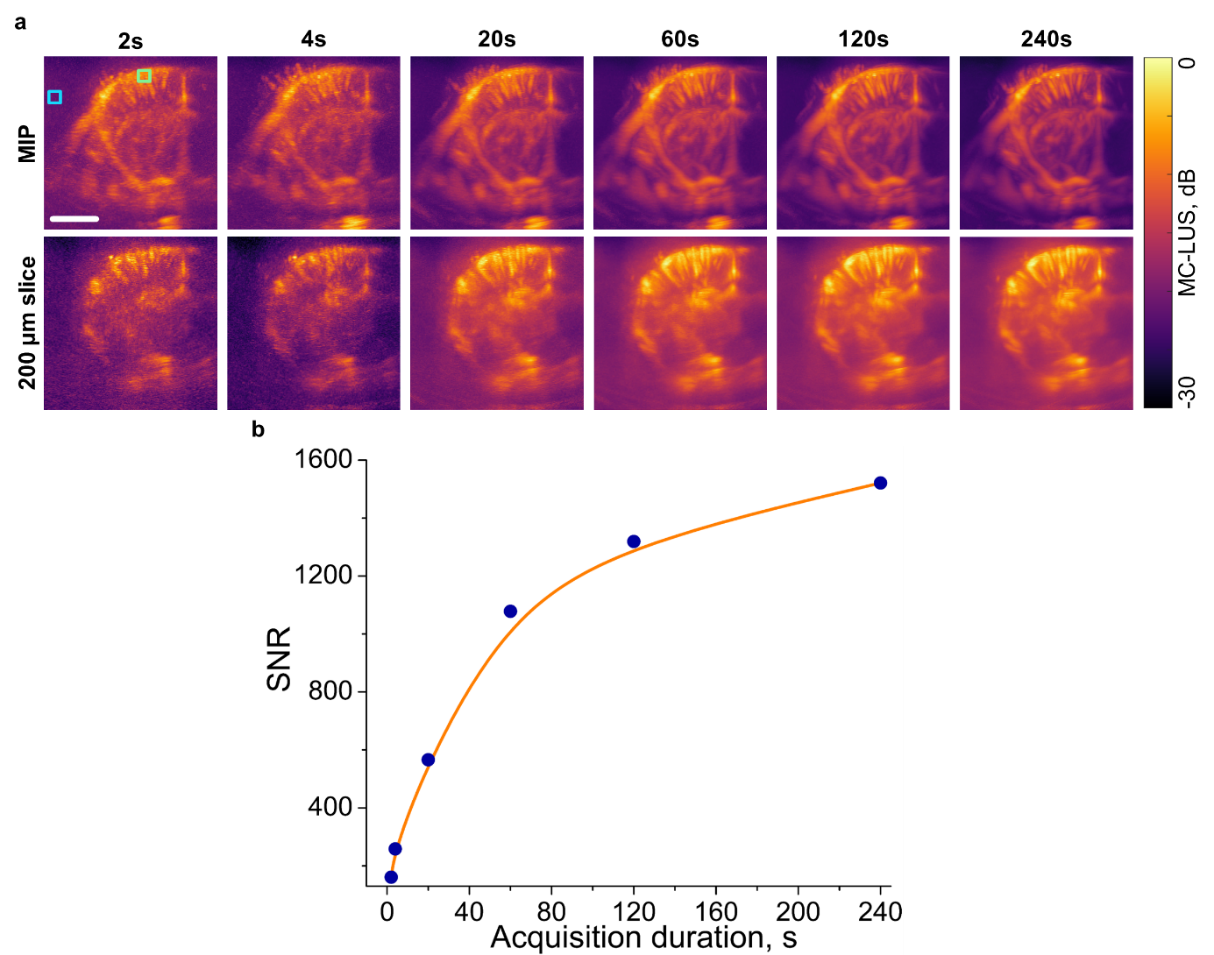
**

**Figure S3.** a) Dependence of image quality on acquisition time considering acquisition of 100 frames per second. Scalebar – 2 mm. b) Signal-to-noise ratio (SNR) calculated according to $SNR= \frac{\mu(Signal)}{\sigma(Noise)}$ based on the highlighted areas in (a): green square – Signal, blue square – Noise. The fitted curve is shown.

**
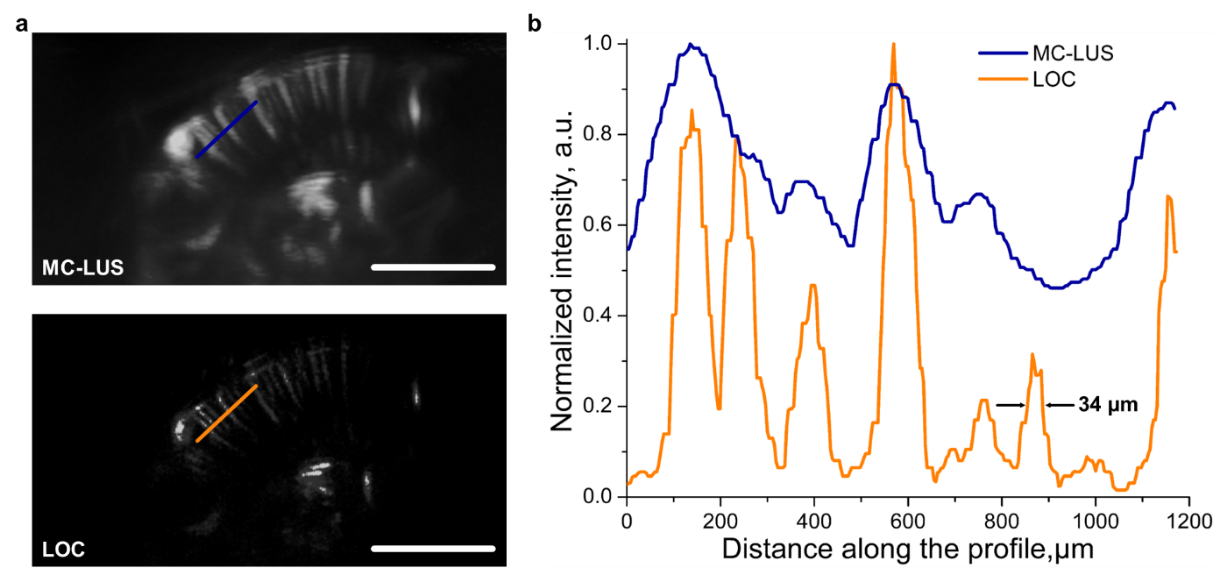
**

**Figure S4.** a) Coronal sections (120 µm thick) of the left hemisphere of the murine brain depicting penetrating vessels in motion contrast laser ultrasound imaging (MC-LUS) and localization (LOC) LUS imaging. Scalebar – 2 mm. b) Profiles perpendicular to the penetrating vessels marked in a. The full-width at half maximum (FWHM) of one of the reconstructed vessels is indicated.

**
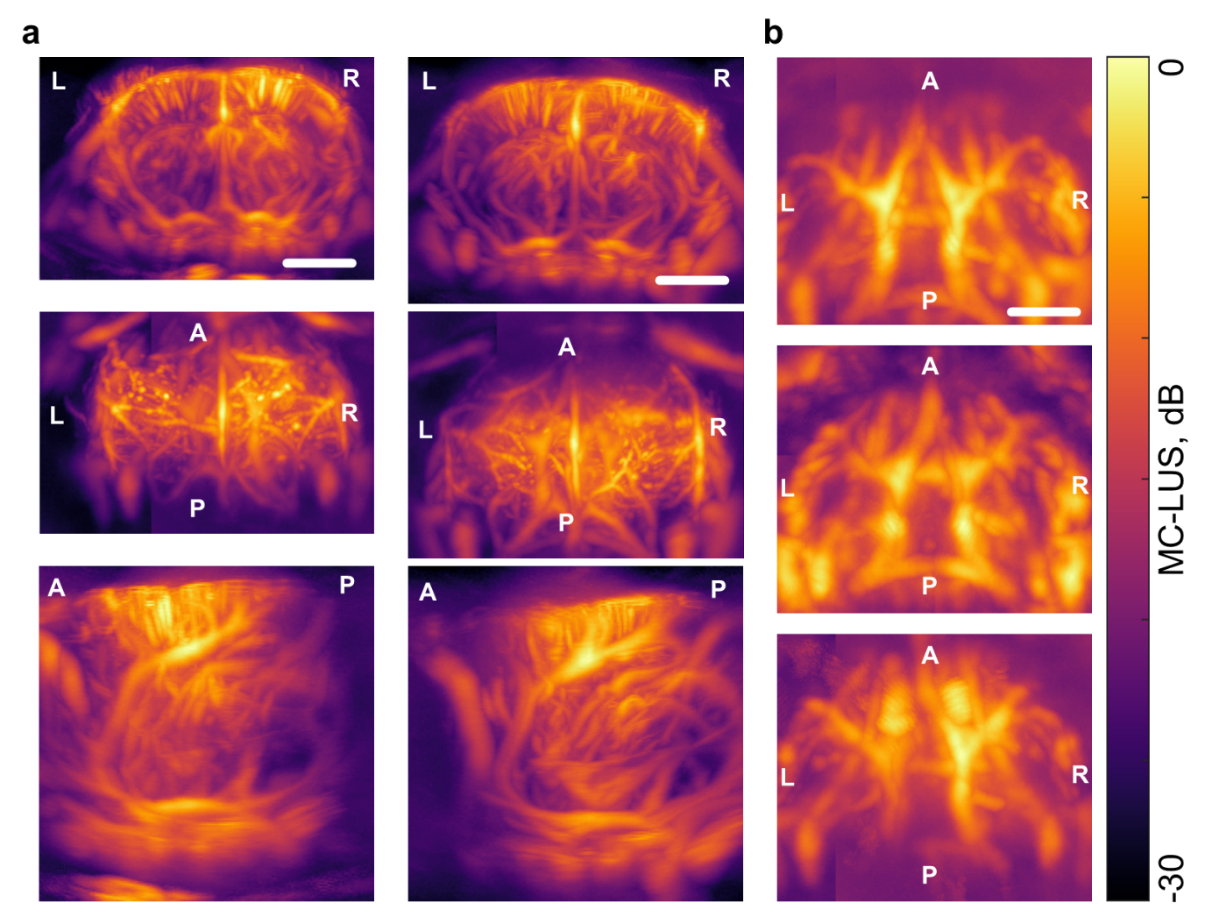
**

**Figure S5.** a) Composed mosaic view of 2 acquisition positions (left and right brain hemispheres) for 2 mice. b) Circle of Willis for 3 different mice. Scalebar - 2mm.


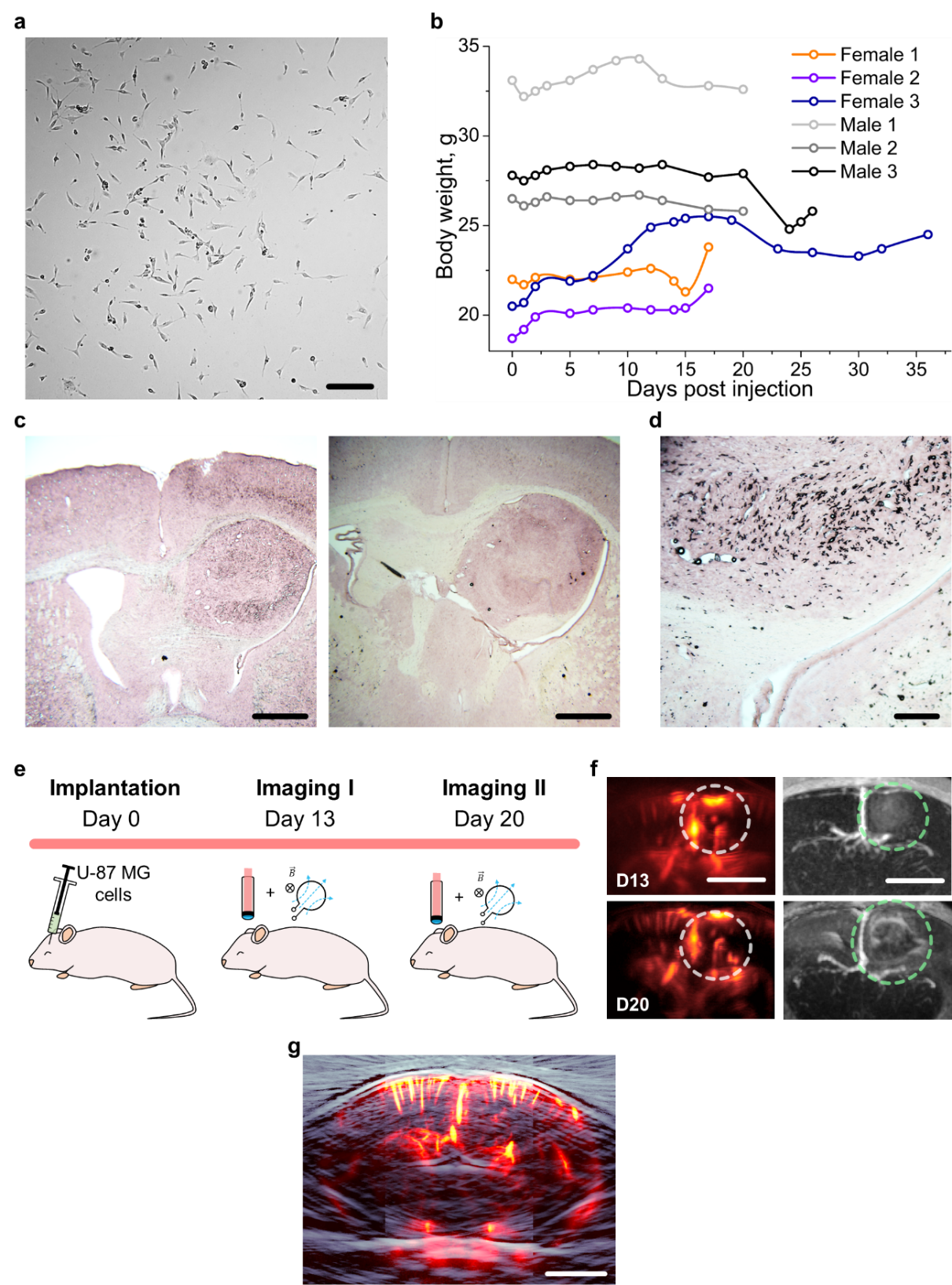


**Figure S6.** a) U-87 MG glioblastoma cell culture. Scalebar – 100 µm. b) Body weight from 6 mice monitoring on different days following injection of tumor cells. c) Hematoxylin-eosin (H&E)-stained histology slices of the brains with the tumor. Scalebar - 1 mm. d) Magnified view of the tumor border. Scalebar – 200 µm. e) Timeline of the longitudinal experiment including two imaging sessions (LUS, OA, and MRA) after inoculation of tumor cells. f) Coronal sections of the LUSSI and MRA images at days 13 and 20 post-inoculation. The tumor location is indicated with dashed circles. g) Combined LUS-LUSSI image of the mouse brain in tumor region. Scalebars – 2 mm.


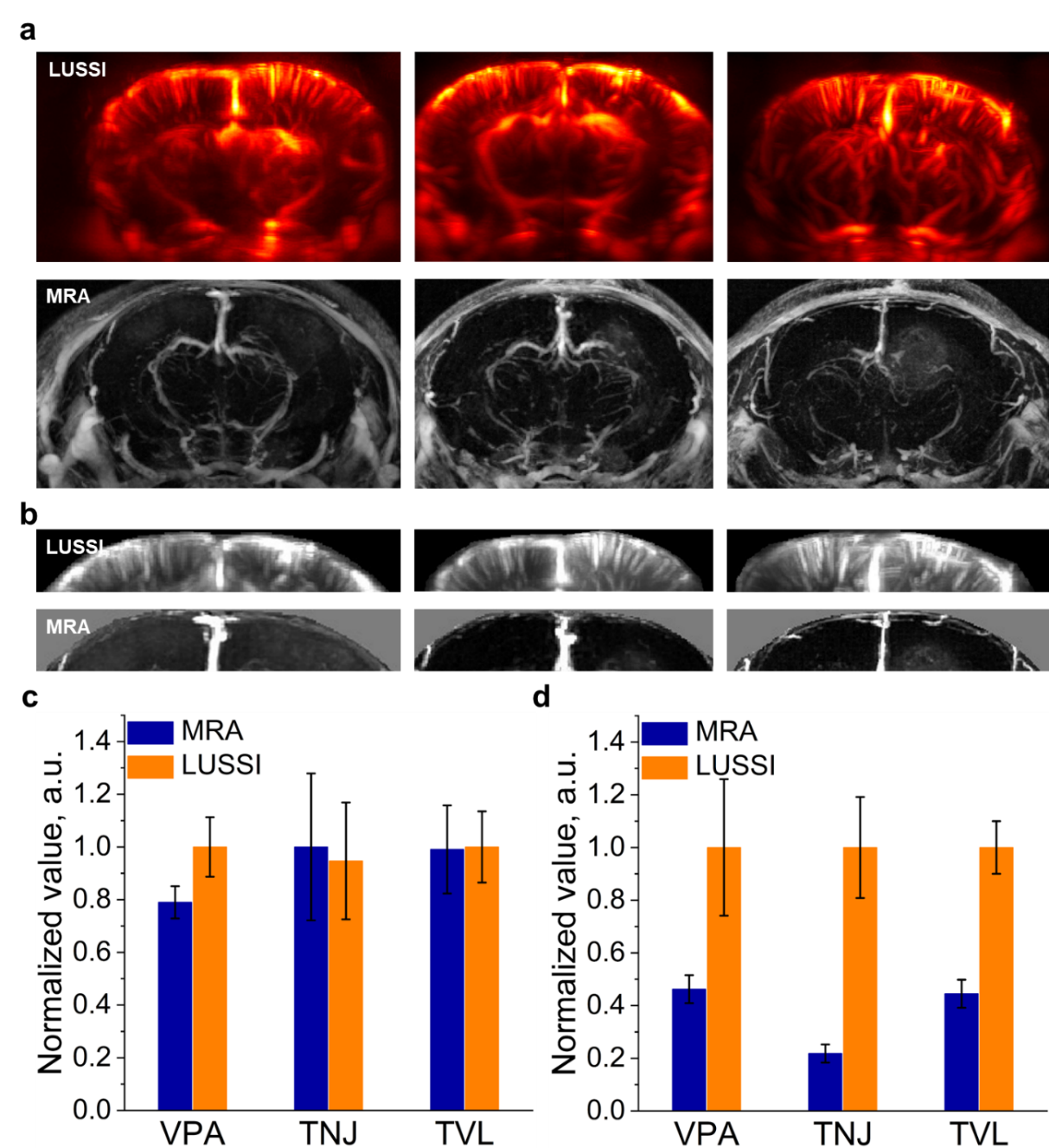


**Figure S7.** a) LUSSI and MRA maximum intensity projections through the tumor volume for 3 different mice: the MRA images, being acquired with resolution 300 µm × 50 µm × 50 µm (X × Y × Z) were linearly upsampled to the reconstruction resolution of LUS 40 µm × 40 µm × 40 µm (X × Y × Z) for comparison. b) Zoom-ins to the cortical region of the same images. c) Microvascular parameters estimated with AngioTool 0.6a across the entire slice (panel a). d) Microvascular parameters estimated with AngioTool 0.6a across the selected cortical area (panel b). VPA – vessel percentage area, TNJ – total number of joints, TVL – total vessel length. For the current parameters, LUSSI provide more information in the cortical region, but MRA matches the score at the larger depths.

**
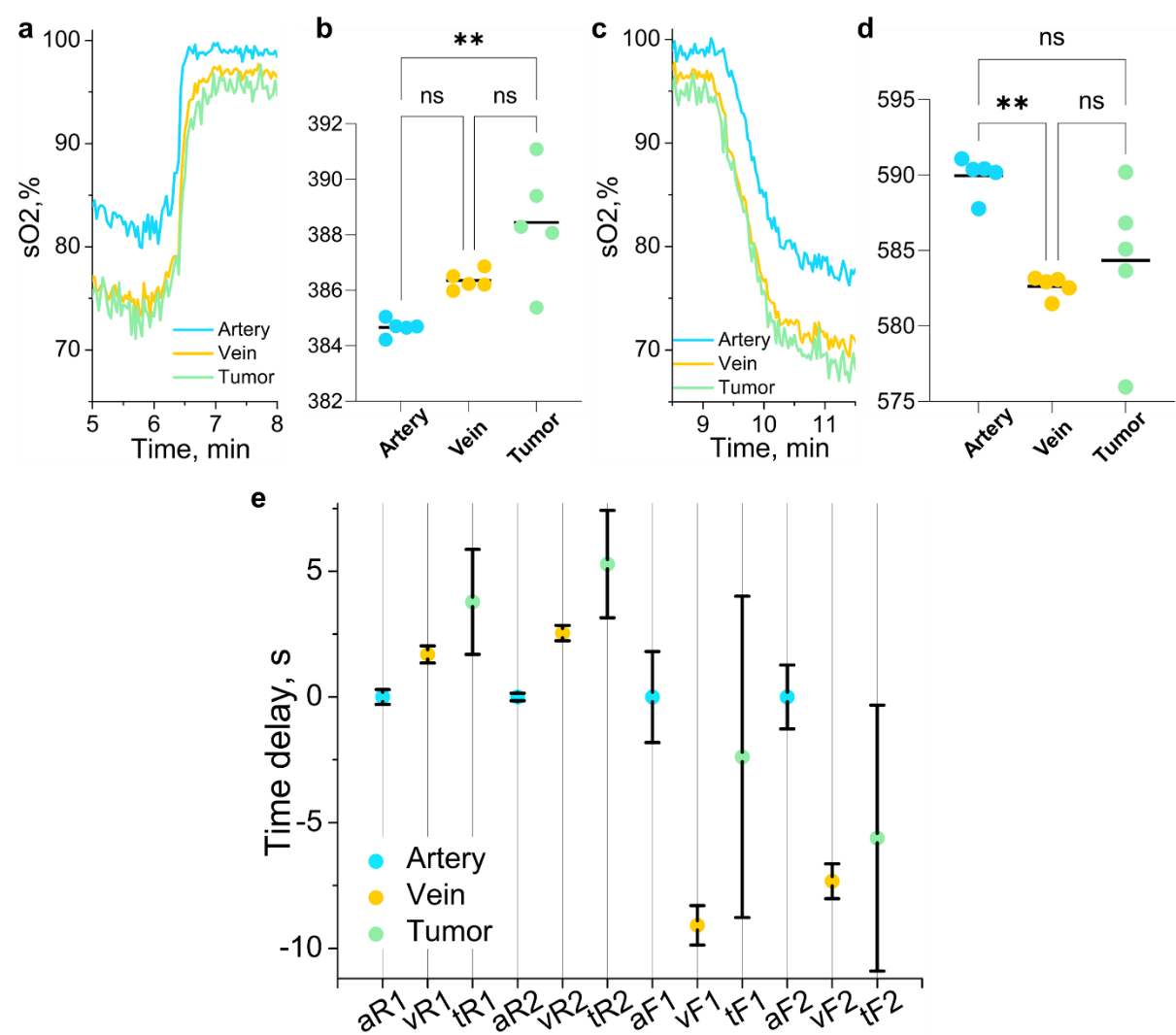
**

**Figure S8.** a) Zoom-in view of the sO2 rise dynamics after the breathing gas change from pure air to pure oxygen. b) Statistical analysis (Kruskal - Wallis test followed by Dunn’s multiple comparison test for 3 groups – artery, vein and tumor rise/fall time, 5 values in each group) results for the rise time, p = 0.0044; c) Zoom-in view of the sO2 fall dynamics after the breathing gas change from pure oxygen to pure air. d) Corresponding statistics on the fall time (p = 0.0071, the statistical test is the same as for the rise time). e) Time delay between the artery (average set to zero), vein, and tumor for the rise times (xR1-xR2) and fall times (xF1-xF2), where x is artery (a), vein (v) or tumor (t).

**Supplementary Videos**

**Video S1.** Rotating view of the laser ultrasound (LUS) image of the tissue-mimicking phantom consisting of 90 µm polyethylene spheres (brown) along with the localized points (green) in a sequence of 2000 frames.

**Video S2.** Rotating view of the murine brain's left hemisphere acquired with the different approaches proposed in this work. OA – optoacoustic tomography; Oxygenation – optoacoustic oxygenation mapping based on the linear unmixing of multispectral optoacoustic tomography data; LUS – laser ultrasound; MC-LUS – motion contrast laser ultrasound; DMC-LUS – directional motion contrast laser ultrasound; LUSSI – laser ultrasound superresolution imaging.

**Video S3.** Rotating view of the 9-position scan of the entire mouse brain: RAW – original image reconstructed with laser ultrasound superresolution imaging (LUSSI). VF – vesselness (Frangi)-filtered image.

**Video S4.** Coronal, sagittal, and transverse cross-sectional views of the entire mouse brain, acquired with 9-position scan laser ultrasound superresolution imaging (LUSSI). The location of the tumor is indicated with a white solid circle. Original and Frangi-filtered images are shown.
